# A virtual sequencer reveals the dephasing patterns in error-correction code DNA sequencing

**DOI:** 10.1093/nsr/nwaa227

**Published:** 2020-09-03

**Authors:** Wenxiong Zhou, Li Kang, Haifeng Duan, Shuo Qiao, Louis Tao, Zitian Chen, Yanyi Huang

**Affiliations:** Biomedical Pioneering Innovation Center (BIOPIC), School of Life Sciences, Beijing Advanced Innovation Center for Genomics (ICG), and Peking-Tsinghua Center for Life Sciences, Peking University, Beijing 100871, China; Biomedical Pioneering Innovation Center (BIOPIC), School of Life Sciences, Beijing Advanced Innovation Center for Genomics (ICG), and Peking-Tsinghua Center for Life Sciences, Peking University, Beijing 100871, China; Biomedical Pioneering Innovation Center (BIOPIC), School of Life Sciences, Beijing Advanced Innovation Center for Genomics (ICG), and Peking-Tsinghua Center for Life Sciences, Peking University, Beijing 100871, China; Biomedical Pioneering Innovation Center (BIOPIC), School of Life Sciences, Beijing Advanced Innovation Center for Genomics (ICG), and Peking-Tsinghua Center for Life Sciences, Peking University, Beijing 100871, China; Center for Bioinformatics, State Key Laboratory of Protein Engineering and Plant Genetic Engineering, Peking University, Beijing 100871, China; Center for Quantitative Biology, Peking University, Beijing 100871, China; Biomedical Pioneering Innovation Center (BIOPIC), School of Life Sciences, Beijing Advanced Innovation Center for Genomics (ICG), and Peking-Tsinghua Center for Life Sciences, Peking University, Beijing 100871, China; College of Engineering, Peking University, Beijing 100871, China; Biomedical Pioneering Innovation Center (BIOPIC), School of Life Sciences, Beijing Advanced Innovation Center for Genomics (ICG), and Peking-Tsinghua Center for Life Sciences, Peking University, Beijing 100871, China; College of Engineering, Peking University, Beijing 100871, China; College of Chemistry and Molecular Engineering, Peking University, Beijing 100871, China; Institute for Cell Analysis, Shenzhen Bay Laboratory, Guangdong 518132, China; Chinese Institute for Brain Research (CIBR), Beijing 102206, China

**Keywords:** DNA sequencing, error-correction code, dephasing, computer simulation, sequencing-by-synthesis

## Abstract

An error-correction code (ECC) sequencing approach has recently been reported to effectively reduce sequencing errors by interrogating a DNA fragment with three orthogonal degenerate sequencing-by-synthesis (SBS) reactions. However, similar to other non-single-molecule SBS methods, the reaction will gradually lose its synchronization within a molecular colony in ECC sequencing. This phenomenon, called dephasing, causes sequencing error, and in ECC sequencing, induces distinctive dephasing patterns. To understand the characteristic dephasing patterns of the dual-base flowgram in ECC sequencing and to generate a correction algorithm, we built a virtual sequencer *in silico*. Starting from first principles and based on sequencing chemical reactions, we simulated ECC sequencing results, identified the key factors of dephasing in ECC sequencing chemistry and designed an effective dephasing algorithm. The results show that our dephasing algorithm is applicable to sequencing signals with at least 500 cycles, or 1000-bp average read length, with acceptably low error rate for further parity checks and ECC deduction. Our virtual sequencer with our dephasing algorithm can further be extended to a dichromatic form of ECC sequencing, allowing for a potentially much more accurate sequencing approach.

## Teaser

A computational model reveals how DNA molecules get unsynchronized in error-correction code sequencing and leads to algorithms to correct the aberrant signals back.

## INTRODUCTION

Next-generation sequencing (NGS) technology has transformed biological and medical research dramatically [1–7]. However, mainstream NGS methods require a DNA amplification step to amplify the signal, causing a phenomenon called ‘dephasing’: within a clone of identical DNA molecules not every molecule is reacting at the same pace, and such asynchronization leads to a mixture of the measurable signals used for sequencing identification. Therefore, dephasing intrinsically limits the accuracy and read length of DNA sequencing. Although single-molecule sequencing may avoid dephasing and can reach an ultra-high read length, the low signal-to-noise ratio and natural stochasticity make it challenging to achieve satisfactory accuracy [8–13]. To date, NGS methods relying on clonal amplification and sequencing are still the major technology in fundamental biology studies and clinical applications. Inventing new chemistry for sequencing DNA is always intriguing but challenging, and dephasing is one of the major problems most new chemistry needs to overcome before reliable sequencing results can be provided.

**Figure 1.**
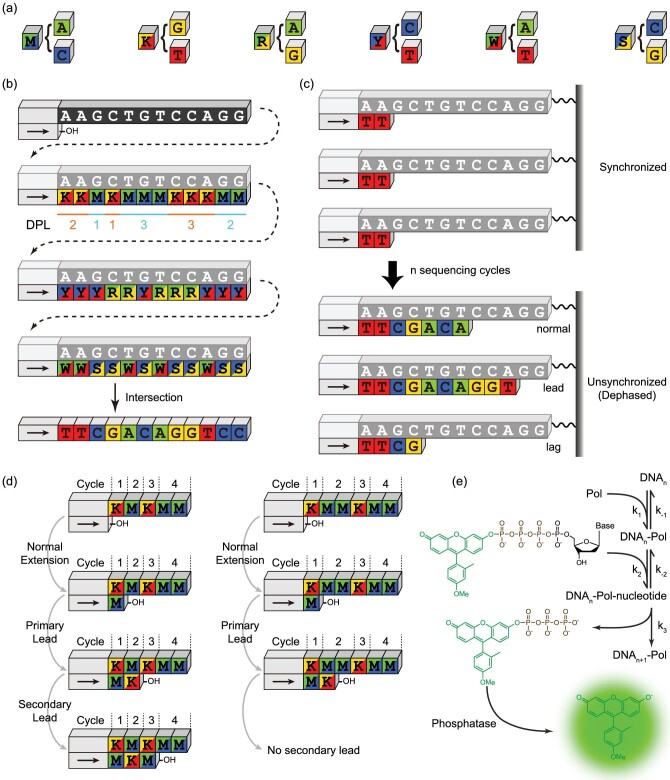
Schematic. (a) The six degenerate bases. (b) Schematic of error-correction code (ECC) sequencing. The ideal sequencing signals, or the lengths of the consecutive identical degenerate bases, is called the ‘degenerate polymer length’ (DPL). (c) The phenomenon that the nascent DNA strands progressively lose their synchronicity, also called ‘dephasing,’ is common in sequencing-by-synthesis-based DNA sequencing technologies. (d) Illustration of ‘one-base slippage’ (OBS) scheme. (e) Chemical reactions simulated in the virtual sequencer.

To reduce DNA sequencing errors originating from the imperfect chemical reactions, we introduced the error-correction code (ECC) concept into sequencing-by-synthesis (SBS) reactions to deduce an unambiguous DNA sequence using three degenerate sequences obtained by a novel dual-base flowgram [14] (Fig. 1a and b). In this approach, we use three orthogonally generated degenerate sequences, which can be considered as binary strings, to perform a parity check in between, and correct the errors through Bayesian probability calculations. The lengths of consecutive identical degenerate bases, the ideal sequencing signals, are called the ‘degenerate polymer length’ (DPL). Although this ECC sequencing technology requires specific unnatural nucleotides as substrates for polymerase to incorporate into the newly synthesized strand, it does hold great potential for providing long sequencing reads while maintaining high accuracy. We noted a unique dephasing pattern exhibited by the specific dual-base flowgram used in ECC sequencing. As an inevitable phenomenon that describes the loss of synchronization (phase) between the DNA extension reactions in a clone of DNA molecules being sequenced (Fig. 1c), dephasing has two components, lead and lag, meaning that the reaction happens in advance or is delayed, respectively. Hence, the true number of DNA nucleotides incorporated in each reaction cycle becomes difficult to determine.

With the exception of single-molecule based approaches, all sequencing chemistry has characteristic dephasing patterns, through which the asynchronous signal can be reconstructed back to DNA sequences [15–20]. The dephasing pattern is a consequence of the nature of sequencing chemistry, reflecting multiple parameters of the reaction, including the yield, the side products, the kinetics and the impurity of the reactional system.

In this article, we present an ordinary differential equation-based model to simulate the clonal reactions of dual-base flowgram of ECC sequencing. This virtual sequencer can faithfully simulate the experimental results of ECC sequencing and identify the major factors that determine the performance of sequencing results, which is majorly a result of the extent of dephasing. Through such simulation we are able to build an effective dephasing algorithm to correct the phases between molecules in a clone, and eventually improve the read length and the raw accuracy before ECC deduction. We also prove that such an algorithm can work well on a dichromatic degenerate sequencing scheme, to enable more accurate sequencing through elevated information provided in each reaction cycle.

## RESULTS AND DISCUSSION

We found that in ECC sequencing the dephasing pattern follows a ‘one-base slippage’ (OBS) scheme, meaning that the signal leading, primarily because of impurity reactions, will easily cause a secondary lead under specific conditions, and exhibits a characteristic dephasing pattern that is fundamentally different from other SBS sequencing chemistries [14]. For example (Fig. 1d), in a typical case where the DNA template to be sequenced is KM*_n_*KMM, the main reactant in the reaction solution is M (A/C) and the impurity is K (G/T). After the first nucleotide K is extended by the main reactant M, the successive M is partially extended by the impurity K (defined as the ‘primary lead’). If *n *= 1, then the second K will be further extended by the excessive main reactant M (defined as the ‘secondary lead’). However, the secondary lead is negligible if *n > *1, because the impurity is a trace amount and will be depleted after the primary lead. Although it has been shown that a practical algorithm for dephasing parameter estimation and signal correction built upon the OBS proposition can be established, it remains unknown when the dephasing correction algorithm is applicable and which factors determine OBS.

To answer these questions, we built a virtual sequencer from first-principles using ordinary differential equations (ODEs) to model the chemical reactions in ECC sequencing (Fig. 1e). The fluorogenic SBS chemistry comprises two major reactions [14,21–23]: the DNA synthesis catalyzed by DNA polymerase (Pol), and the dephosphorylation catalyzed by the alkaline phosphatase. In our model the dephosphorylation can be omitted because it is a much faster reaction compared with DNA synthesis.

By setting the concentrations of the four fluorogenic nucleotides in each cycle, we are able to numerically simulate the sequencing process with any desired flowgram, including the dual-base flowgram used in ECC sequencing and, for comparison, the conventional single-nucleotide addition flowgram. Taking the MK flowgram in ECC sequencing as an example, we add excessive M as the main species and trace amounts of K as the impurity in every odd cycle, and excessive K and trace amounts of M in every even cycle. The final values of the fluorophore *F* are regarded as the fluorescent intensities detected in each cycle, and *d*[*F*]/*dt *= *k*_3_[*P* · *D_k__ _*− 1 · *N_k_*], where *D_k_* stands for the *k*-bp primed DNA strand, and *N_k_* stands for the nucleotide complementary to the *k*-th base of the template.

Using our virtual sequencer and by tuning the impurity amount and reaction time, we simulated four typical sequencing conditions, with and without lead or lag, in all combinations. If the SBS reaction for all of the DNA molecules is perfectly synchronous, there will be no lead or lag, the fluorescent signals produced in each cycle will be proportional to the length of each copolymer, and all primed DNA strands will have exactly the same length (Fig. S1a and b). When the reaction loses its synchronicity, the fluorescent signals become aberrant, and there will be lead and/or lag, resulting in an increasing amount of primed DNA strands leading forward or lagging behind the main primed DNA (Fig. S1c–h). It is clear that the lead is mainly caused by impurities in the reaction buffer, and the lag is primarily a result of insufficient reaction time within which some of the molecules do not finish the reaction.

We first simulated the single-cycle sequencing using dual-base flowgram on the sequence A(G)*_n_*AAA(*n *= 1*,*2*,*3). The main nucleotide species is T and the impurity is C. In the simulation, we sampled six parameters 10 000 times in the range given in Column 2, Table S1. Among the six sampling parameters, the concentrations of the impurity and Pol, and the reaction time are sampled uniformly, while the reaction rate constants *k*_1_, *k*_2_ and *k*_3_ are sampled logarithm uniformly (Table S1). We also fixed *k*_−1_ = 0*.*01*k*_1_ and *k *_−2_ = 10*k*_2_ according to reported chemical equilibrium constants [24–26].

**Figure 2.**
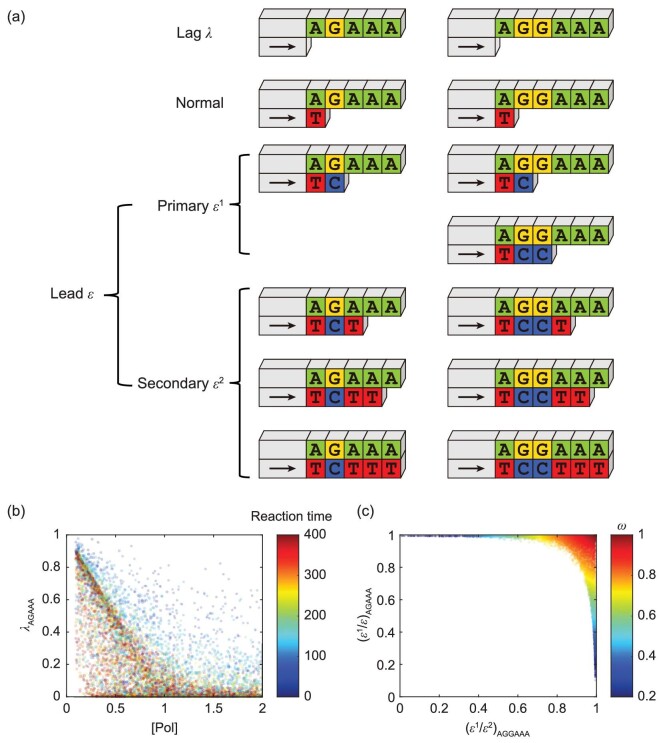
Key factors of dephasing patterns revealed by single-cycle simulation. (a) Dephasing parameters and their corresponding nascent DNA strands. (b) Insufficient Pol will cause severe lag, which can be remedied by prolonged reaction time. (c) Distribution of *ϵ*^1^ percentage in AGGAAA and *ϵ*^2^ percentage in AGAAA of each set of parameters, which gives the definition of the OBS index.

After sequencing, the primed DNA is extended to different lengths, and we use an all lowercase sequence to denote the unextended primed DNA (e.g. agaaa), an uppercase letter in the sequence to denote the primed DNA extending to the position of the uppercase letter (e.g. aGaaa) and multiple upper case letters in the sequence to denote the sum of primed DNA denoted by a single upper case (e.g. agAAA = agAaa + agaAa + agaaA). For A(G)*_n_*AAA, the dephasing parameters lag (*λ*), primary lead (*ϵ*^1^) and secondary lead (*ϵ*^2^) can be defined as [a(g)*_n_*aaa], [a(G)*_n_*aaa] and [a(g)*_n_*AAA], respectively. We also define the total lead *ϵ* = *ϵ*^1^ + *ϵ*^2^ (Fig. 2a).

The *λ* and *ϵ* measured in the three sequences, AGAAA, AGGAAA and AGGGAAA, are very close to each other, especially when the values are small (Figs S2 and S3). Not surprisingly, except for *k*_2_ and *λ*, other sampling parameters have significant correlations with the dephasing parameters in terms of Spearman's correlation coefficient (Figs S4–S7). However, empirically, the most notable correlations are: 1) *ϵ* is equivalent to the impurity concentration when *λ* is small; 2) insufficient Pol will cause severe lag effect, but it becomes less important after saturating the DNA template (Fig. 2b). Additionally, the *ϵ*^1^ in AGGAAA and the *ϵ*^2^ in AGAAA are tightly correlated (Fig. S8).

If OBS holds, then the major contribution of *ϵ* in AGGAAA and AGAAA should be *ϵ*^1^ and *ϵ*^2^, respectively, as verified in Fig. 2c. To quantitatively assess how OBS approximates the simulation results of the virtual sequencer, we define the OBS index *ω* = (*ϵ*^1^/*ϵ*)_AGGAAA_ · (*ϵ*^2^/*ϵ*)_AGAAA_, a parameter that shows significant correlation to all sample parameters (Figs S9 and S10). Notably, a greater *ϵ* or *λ* causes a lower *ω*, indicating that an optimized sequencing protocol with less dephasing is desired (Fig. S11).

We selected *ω* ≥ 0*.*99 as the criterion when OBS holds. Among the 10 000 sets of sampling parameters, 138 sets satisfy *ω* ≥ 0*.*99 with impurity concentration limited within 0.02 and *k*_3_ > 0.1 (Table S1 and Fig. S12). Highly correlated with impurity concentration, the lead is also limited within 0.02. Lag < 0.02 is preferred, but lag as great as 0.78 is also possible to satisfy *ω* ≥ 0*.*99 (Fig. S13).

To validate the parameter range such that *ω* ≥ 0*.*99, we performed second round single-cycle sequencing simulation. The second-round simulation was the same as the first round except for the parameter sampling range (Column 3, Table S1) being narrower. Similar distributions of lead and lag and parameter correlations were observed (Figs S14–S20). However, only 5242 out of 10 000 sets of parameters satisfy *ω* ≥ 0*.*99 with no significant shrink in range (Figs S21 and S22) or correlation in between (Fig. S23). Two extreme parameter subsets exist that help us further clarify the desynchronization mechanism of the dual-base flowgram (Figs S24 and S25). The first subset has a large *λ* (> 0*.*1), but OBS still holds. In this subset, a lack of Pol and a small *k*_1_ account for the large lag, but even less impurity and a relatively large *k*_3_ ensure *ω* >* *0*.*99. The second subset is the opposite: *λ* <* *0*.*01 but *ω* <* *0*.*97. This is mainly a result of the low percentage of *ϵ*^2^ in AGAAA, which can be explained by the limited reaction time and *k*_3_. Overall, the impurities and *k*_3_ seem to be the main determinants of *ω*, while the value of *λ*, mainly determined by the Pol concentration, the reaction time and *k*_1_, is preferred to be small, but is not determinant for *ω*.

We simulated a 100-cycle MK flowgram with our virtual sequencer, and found that our OBS scheme could approximate the DNA length distribution fairly well. The sequences were picked from the *E. coli* genome with a length containing exactly 110 DPLs (Table S2). All four DNA templates were sequenced under the same 1000 sets of parameters, among which the impurity concentration and reaction time are uniformly sampled in the range of [0, 0.1] and [10, 100], respectively, while the rest parameters are set to default. The dephasing parameter estimation and signal correction of the simulated sequencing signals were performed using our previous algorithm [14]. To test whether the lead and lag were estimated accurately, we also simulated the single-cycle sequencing of AGAAA using the same parameters in the 100-cycle simulation. The lead *ϵ* and lag *λ* are measured as [aGAAA] and [agaaa]. Fig. S26 shows that the estimated lead and lag in the 100-cycle simulation are very close to their measured values in the single-cycle simulation, especially when they are small.

**Figure 3.**
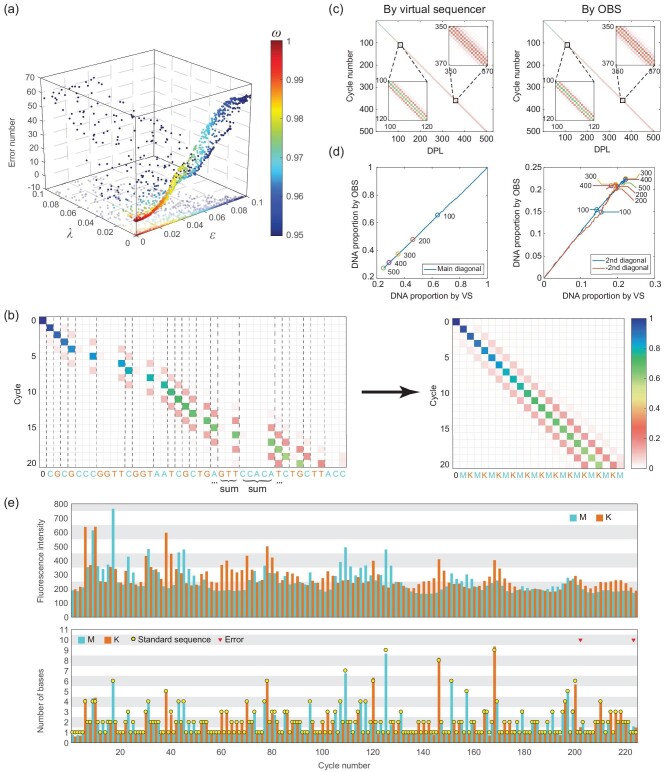
The DPL distribution matrix and dephasing correction. (a) Relationship between *ϵ*, *λ*, *ω* and error number in the dephasing correction of 100-cycle simulated sequencing signals. (b) The base distribution matrix is transformed into the DPL distribution matrix through summing up columns in the same DPL. (c) The DPL distribution matrix per cycle obtained through two methods using the same colormap in (b). (d) Comparison of the main diagonal (left) and ±2nd diagonal (right) of the DPL distribution matrix per cycle obtained through two methods. Numbered dots indicate cycle numbers. VS, virtual sequencer. (e) Fluorescent signals in an ECC sequencing experiment (top) and their dephasing-corrected signals (bottom).

The overall performance of our dephasing correction can be simply indicated using an error number, which is defined as the number of different DPLs between the result c (the DPL after OBS-based dephasing correction) and the true DPL h (the known input for virtual sequencing). We confirmed that as *ω* increases, all lead, lag and error numbers of dephasing corrections decrease (Figs S26–S29). When *ω > *0*.*99, the error number is limited to 5, which is sufficient for accurate ECC sequencing. Moreover, as expected, a large *ϵ* or *λ* causes a high error rate (Fig. 3a). As we observed in the virtual sequencing of AGAAA, dephasing correction simulation shows many identical trends; for example many correlations exist between parameters, *ϵ* is determined by impurity concentration, *λ* is determined by reaction time, and a large *ϵ* or *λ* leads to the decrease of *ω* (Fig. S26).

We further simulated 250-cycle and 500-cycle sequencing scenarios, using sequences from the *E. coli* genome, at the same genomic location as one of the previous 100-cycle simulations (Table S2). Only one error remained after dephasing correction in the 250-cycle simulation, which occurred in the 249th cycle (Figs S30 and S31). Five errors occurred in the 500-cycle simulation, initially at the 335th cycle (Fig. S32). The DPL distribution matrix, which illustrates the loss of reaction synchronization by dephasing, can also be obtained by either fitting the dephasing correction algorithm, or by direct calculations of the ODE-based virtual sequencer result (Fig. 3b). We found that these two methods yield highly consistent DPL distribution matrices (Fig. 3c and d, Fig. S33), showing that the OBS principle can support accurate dephasing correction for long sequencing signals of at least 500 cycles (1000 bp on average).

We then used our experimental prototype to sequence a DNA template for 224 cycles (read length 451 bp) using the MK flowgram, and tested the OBS-based dephasing correction. We found only two errors, one in the 202nd cycle and the other in the 223rd cycle, indicating that the first 410 bp of sequencing is error-free (Fig. 3e).

**Figure 4.**
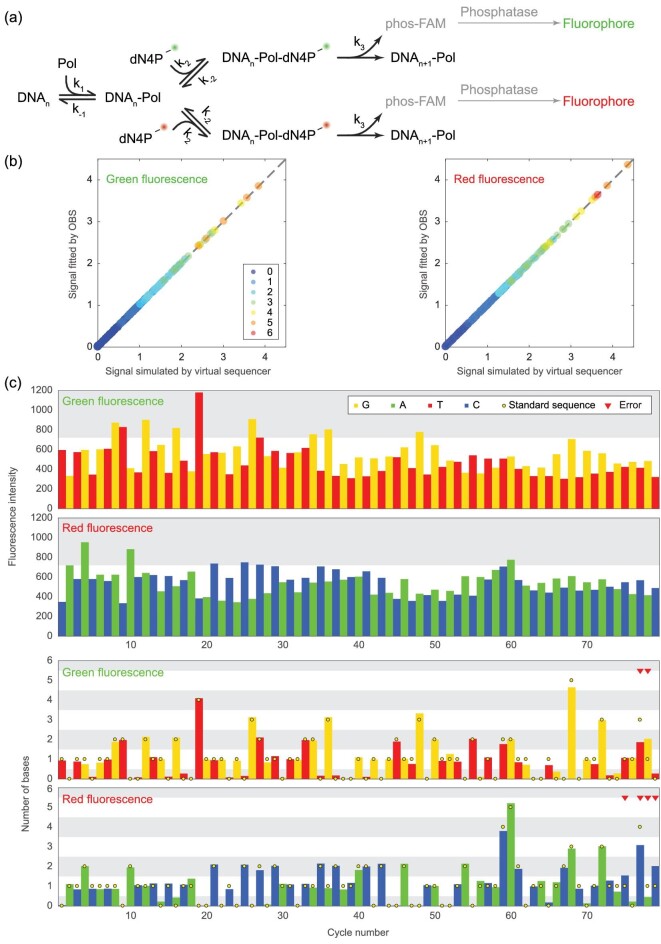
Dichromatic dual-base flowgram simulated by the virtual sequencer. (a) Dichromatic fluorogenic SBS reactions. (b) The signals by the flux matrix fit well with the simulated signals by the dichromatic virtual sequencer. Color denotes the DPL. (c) The fluorescence intensities (top) and their dephasing corrected signals (bottom) of a dichromatic ECC sequencing experiment.

So far, our discussion has been based on a monochromatic dual-base flowgram, meaning that the two nucleotides in one degenerate reaction cycle are labeled with the same fluorophore, whose intensity reveals the total number of nucleotides incorporated in a cycle. In fact, the virtual sequencer simulator can be further extended to a dichromatic mode, where the two nucleotides in one reaction cycle are labeled with different fluorophores. For example, in the MK flowgram, A and C are added in every odd cycle and G and T are added in every even cycle, while A and G are labeled with a green dye and C and T are labeled with a red dye (Fig. 4a). Thus, we would be able to separately measure the number of each nucleotide that is extended in any given cycle. Dichromatic ECC sequencing provides 3.37 bit/cycle of information, which is much higher than the 2 bit/cycle afforded by monochromatic ECC sequencing and therefore has the potential to be even more accurate. Because there are two different dyes labeled for the four nucleotides, the sizes of DPL **h** and fluorescent intensities **f** change from *m *× 1 and *n *× 1 to *m *× 2 and *n *× 2, respectively. To correct the dephased dichromatic signals, we hypothesize that the basic equation **f **= *T***h** in monochromatic dephasing still holds. Specifically, **f**_•1_ = *T***h**_•1_, and **f**_•2_ = *T***h**_•2_.

To validate this hypothesis, we simulated the dichromatic ECC sequencing for 250 cycles, using the same parameters as in the 250-cycle monochromatic simulation (Fig. S34). The DPL distribution matrix *D* is given directly by the virtual sequencer, while the flux matrix *T* is deduced from *D* by:

$$\begin{equation*}
{T_{i,j}} = \left\{ {\begin{array}{@{}*{2}{l}@{}} {{D_{i,j}} - {D_{i - 1,j}}}&{j = m}\\ {{T_{i,j + 1}} + {D_{i,j}} - {D_{i - 1,j}}}&{j < m} \end{array}} \right.
\end{equation*}$$

We compared the simulated fluorescent signals **f** and the signals fitted by *T***h** and found the signals to be almost identical (Fig. 4b), thus confirming our hypothesis, and suggesting that our OBS-based dephasing correction algorithm can be directly applied to the dichromatic mode. To experimentally validate this suggestion, we sequenced a DNA template for 79 cycles (read length 165 bp) using the dichromatic MK flowgram and corrected the fluorescent signals (Fig. 4c). The first error appeared at the 75th cycle, indicating that the first 152 bp were error-free.

## CONCLUSION

In summary, we constructed a virtual sequencer to simulate fluorogenic SBS sequencing reactions, and found that the characteristic ‘one-base slippage’ dephasing pattern can be applied to correct for dephasing. Using our virtual sequencer, we clarified the dephasing mechanism of ECC sequencing, and extended our understanding to the dichromatic form of ECC sequencing. Our virtual sequencer also revealed that, when applying to dichromatic instead of monochromatic ECC sequencing, the relationship (involving matrix multiplication) linking the DPL and sequencing signals remains unchanged.

The OBS pattern is common in ECC sequencing. However, it also exists in traditional single-nucleotide addition (SNA) sequencing methods such as pyrosequencing [27] and semiconductor sequencing [28], although at a lower frequency. The impurities in SNA sequencing are mainly the nucleotides left over from the previous cycle, thus OBS may occur in DNA motifs X_m_Y_n_XY_k_. Allowing for OBS in dephasing algorithms of SNA sequencing may also improve their accuracy.

Stochastic models are widely used in systems biology and noise plays an indispensable role in many biochemical processes [29–34]. However, the stochastic version of the virtual sequencer showed negligible difference to the ODE-based deterministic version (data not shown). That is because there are typically >5 × 10^3^ DNA molecules and even more enzymes and nucleotides in the sequencing reaction, which greatly reduce the reaction noise. Besides, ECC sequencing chemistry comprises only two consecutive enzymatic reactions and noise is unlikely to play a role in such a simple reaction topology.

Understanding dephasing patterns in ECC sequencing not only provides insights into SBS reactions, but also suggests that our approach can lead to the design and further optimization of dephasing algorithms in general. For example, the fluorescent signals of some DNA reads are incomplete because of occasional chip contaminants or sight offset. The virtual sequencer may be used to simulate abnormal sequencing signals and hence aid the development of a special dephasing algorithm.

## METHODS

Based on mass-action, the ODEs in the virtual sequencer are

$$\begin{equation*}
\left\{ {\begin{array}{@{}*{1}{l}@{}} {\frac{{d\left[ P \right]}}{{dt}} = {k_{ - 1}}\left[ {P \cdot {D_{k - 1}}} \right] - {k_1}\left[ P \right]\left[ {{D_{k - 1}}} \right]}\\
{\frac{{d\left[ {{D_{k - 1}}} \right]}}{{dt}} = {k_{ - 1}}\left[ {P \cdot {D_{k - 1}}} \right] - {k_1}\left[ P \right]\left[ {{D_{k - 1}}} \right]}\\
{\frac{{d\left[ {P \cdot {D_0}} \right]}}{{dt}} = {k_1}\left[ P \right]\left[ {{D_0}} \right] - {k_{ - 1}}\left[ {P \cdot {D_0}} \right] + {k_{ - 2}}\left[ {P \cdot {D_0} \cdot {N_1}} \right] - {k_2}\left[ {P \cdot {D_0}} \right]\left[ {{N_1}} \right]}\\
{\frac{{d\left[ {P \!\cdot\! {D_k}} \right]}}{{dt}} = {k_1}\left[ P \right]\left[ {{D_k}} \right] - {k_{ - 1}}\left[ {P \!\cdot\! {D_k}} \right] + {k_{ - 2}}\left[ {P \!\cdot\! {D_k} \!\cdot\! {N_{k + 1}}} \right] - {k_2}\left[ {P \!\cdot\! {D_k}} \right]\left[ {{N_{k + 1}}} \right] + {k_3}\left[ {P \!\cdot\! {D_{k - 1}} \!\cdot\! {N_k}} \right]}\\
{\frac{{d\left[ {{N_k}} \right]}}{{dt}} = {k_{ - 2}}\left[ {P \cdot {D_{k - 1}} \cdot {N_k}} \right] - {k_2}\left[ {P \cdot {D_{k - 1}}} \right]\left[ {{N_k}} \right]}\\
{\frac{{d\left[ {P \cdot {D_{k - 1}} \cdot {N_k}} \right]}}{{dt}} = {k_2}\left[ {P \cdot {D_{k - 1}}} \right]\left[ {{N_k}} \right] - {k_{ - 2}}\left[ {P \cdot {D_{k - 1}} \cdot {N_k}} \right] - {k_3}\left[ {P \cdot {D_{k - 1}} \cdot {N_k}} \right]}\\
{\frac{{d\left[ F \right]}}{{dt}} = {k_3}\left[ {P \cdot {D_{k - 1}} \cdot {N_k}} \right]} \end{array}} \right.
\end{equation*}$$

where $P$ stands for Polymerase (Pol), ${D_k}$ stands for the k-bp primed DNA strand, ${N_k}$ stands for the nucleotide complementary to the k-th base of the template, and $F$ for the fluorophore. In one run of the virtual sequencer, the ODE is serially solved numerically for many cycles. In Cycle 1, the virtual sequencer sets the initial value of ${D_0}$ to 1 and ${D_k}$ to 0. And in Cycle *i *+ 1, the initial values of ${D_0}$ and ${D_k}$ are set to their final values in Cycle *i*. The final values of $F$ in each cycle are regarded as the detected fluorescent intensities (signal). These settings are followed in this article if there is no additional explanation: the concentration of Pol is 1, the concentration of the main species is 30, ${k_1}$ = ${k_2}$ = ${k_3}$ = 1, ${k_{ - 1}}$ = 0.01, ${k_{ - 2}}$ = 10.

The dephasing parameter estimation and signal correction of the simulated sequencing signals are done using a simplified version of the algorithm described in Ref. [14]. Specifically, for DPL ${{\bf{h}}_{m \times 1}}$ ($m$ = 110 denotes the DPL number) and fluorescent intensities ${{\bf{f}}_{n\times 1}}$ ($n\,\,$= 100 denotes the cycle number) simulated by the virtual sequencer, we calculate the DPL distribution matrix $D$ and flux matrix $T$ as functions of lead and lag:

$$\begin{equation*}
{D_{i,j}} \!=\! \left\{ {\begin{array}{@{}*{2}{c}@{}} 1&{i = j = 1}\\ 0&{i = 1,j \!>\! 1}\\ {{D_{i - 1,j}} - {T_{i - 1,j}}}&{j = 1}\\ {{D_{i - 1,j}} - {T_{i - 1,j}} + {T_{i - 1,j - 1}}}&{j > 1} \end{array}} \right.
\end{equation*}$$

$$\begin{equation*}
{T_{i,j}} = \left\{ {\begin{array}{@{}*{2}{cl}@{}} {\left( {1 - \lambda } \right)\!{D_{i,j}}}&\quad\!\!\!{j \!=\! 1,\left( {i\! + \!j} \right)} \\
&\quad\,\,{\!\equiv\! 0\left( {{\rm{mod}}2} \right)}\\ 0&\quad\!\!\!{j \!=\! 1,\left( {i \!+\! j} \right)} \\
&\quad\,\,{ \equiv 1\left( {{\rm{mod}}2} \right)}\\ {\left( {1 - \lambda } \right){D_{i,j}} + h^{\prime}{T_{i,j - 1}}}&\quad\!\!\!{j \!>\! 1,\left( {i \!+\! j} \right) } \\
&\quad\,\,{\!\equiv\! 0\left( {{\rm{mod}}2} \right)}\\ {\varepsilon \left( {1 - \lambda } \right){D_{i,j - 1}}}&\quad\!\!\!{j \!>\! 1,\left( {i \!+\! j} \right)} \\
&\quad\,\,{ \!\equiv\! 1\left( {{\rm{mod}}2} \right)} \end{array}} \right.
\end{equation*}$$

where $\lambda $ and $\varepsilon $ are lag and lead coefficients to be estimated, respectively, and

$$\begin{equation*}
h_i^{\prime} = \left\{ {\begin{array}{@{}*{2}{c}@{}} 0&\quad{{h_i} > 1}\\ 1&\quad{{h_i} \le 1} \end{array}} \right.\,\,
\end{equation*}$$

The DPL distribution matrix $D$ is used to describe how the primed DNA is dephased during the sequencing, and the flux matrix $T$ links the DPL ${\bf{h}}$ and fluorescent intensities ${\bf{f}}$ through the basic equation:

$$\begin{equation*}
{\bf{f}} = T{\bf{h}}
\end{equation*}$$

Hence, we estimate $\lambda $ and $\varepsilon $ from ${\bf{h}}$ and ${\bf{f}}$ by:

$$\begin{equation*}
\mathop {\min }\limits_{\lambda ,\varepsilon } ||T{\bf{h}} - {\bf{f}}|{|_2}.
\end{equation*}$$

And the corrected signals are calculated as:

$$\begin{equation*}
{\bf{c}} = {T^ + }\,\,{\bf{f}}
\end{equation*}$$

where ${T^ + }$ is the Moore-Penrose pseudoinverse of $T$.

## Supplementary Material

nwaa227_Supplemental_File
