# Supplementary material for: A virtual sequencer reveals the dephasing patterns in error-correction code DNA sequencing
Source: Natl Sci Rev. 2020 Sep 3;8(5):nwaa227. doi: 10.1093/nsr/nwaa227 (PMC8288425; doi:10.1093/nsr/nwaa227)
Supplement: nwaa227_Supplemental_File [file nwaa227_supplemental_file.pdf]

Supplementary Information
A Virtual Sequencer Reveals the Dephasing Patterns in Error-Correction Code DNA sequencing

Wenxiong Zhou, Li Kang, Haifeng Duan,
Shuo Qiao, Louis Tao, Zitian Chen, and Yanyi Huang

July 16, 2020

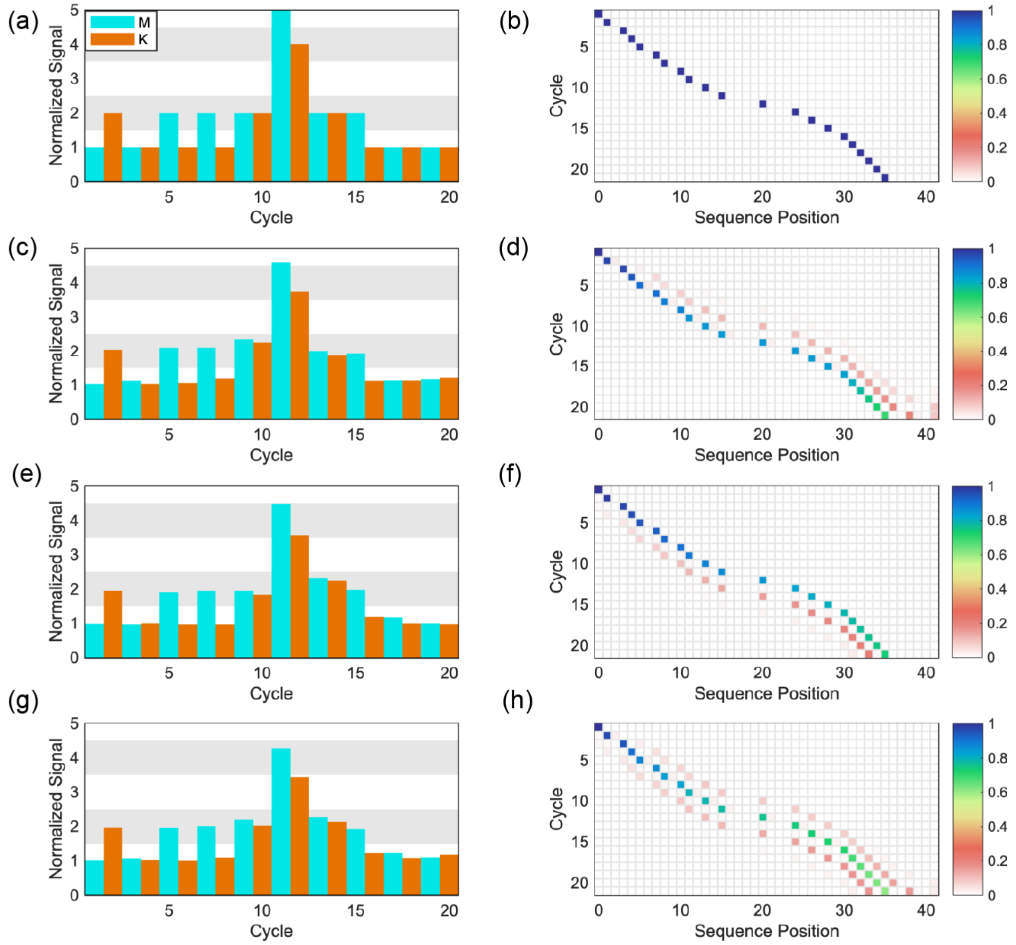

Figure S1. Schematic of the simulated chemical reactions. The DNA template used in this simulation is Seq2. (aceg) Simulated sequencing signals. (bdfh) Simulated DNA length distributions. (ab) Impurity: 0; reaction time: 100. (cd) Impurity: 0.03; reaction time: 100; (ef) Impurity: 0; reaction time: 30. (gh) Impurity: 0.03; reaction time: 30.

Table S1. Virtual sequencer parameter range such that ω>0.99.

Parameters	Overall Range	ω>0.99 Range	ω>0.99 Mean	ω>0.99 Median
Impurity	[0,0.5]	[0.0002,0.02]	0.0082	0.0074
Reaction Time	[50,500]	[55.7,499.2]	324.2	337.5
Polymerase Concentration	[0.1,2]	[0.1281,1.9991]	1.2124	1.303
$\log_{10} k_1$	[-2,1]	[-1.9997,0.9998]	-0.2717	-0.2374
$\log_{10} k_2$	[-2,1]	[-1.9940,0.9941]	-0.4863	-0.5775
$\log_{10} k_3$	[-2,1]	[-1.0077,0.9995]	0.0689	0.0981
lead in AGAAA	[8.8e-06,0.50]	[0.0002,0.0197]	0.0081	0.0070
lead in AGGAAA	[8.8e-06,0.40]	[0.0002,0.0195]	0.0081	0.0070
lead in AGGGAAA	[8.8e-06,0.40]	[0.0002,0.0195]	0.0081	0.0070
lag in AGAAA	[0,0.97]	[0,0.7780]	0.0471	3.5e-10
lag in AGGAAA	[0,0.97]	[0,0.7776]	0.0469	9.0e-10
lag in AGGGAAA	[0,0.97]	[0,0.7776]	0.0469	9.2e-10

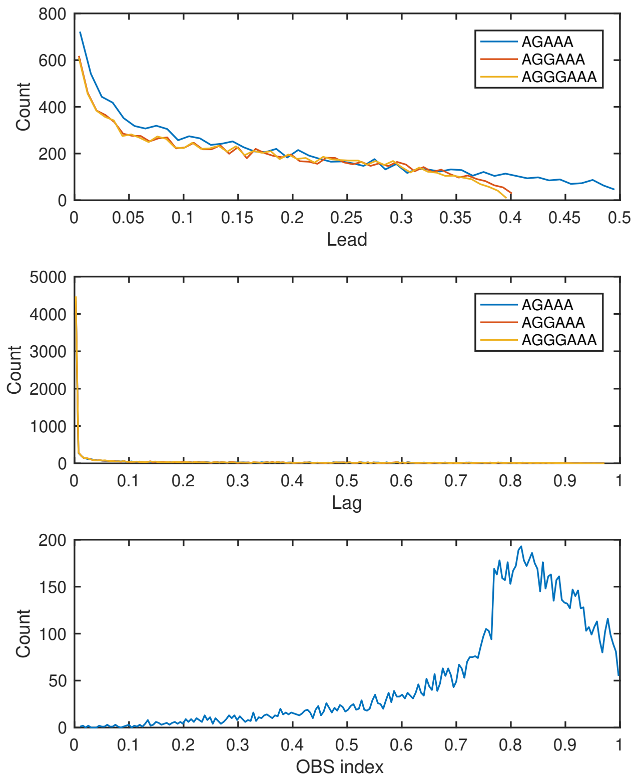

Figure S2. Distribution of total lead (top), total lag (middle) and OBS index (bottom).

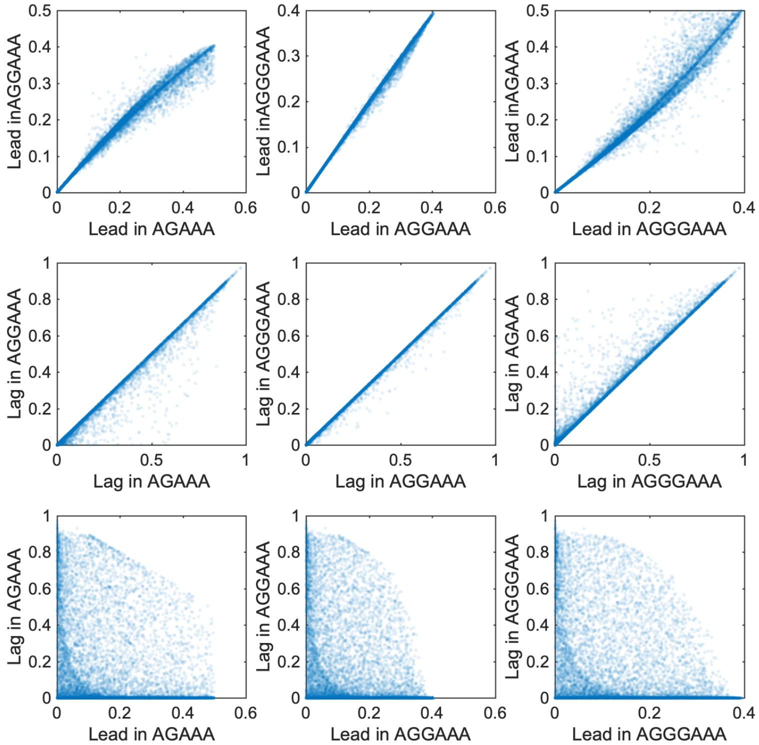

Figure S3. Correlation between the dephasing parameters in different sequences.

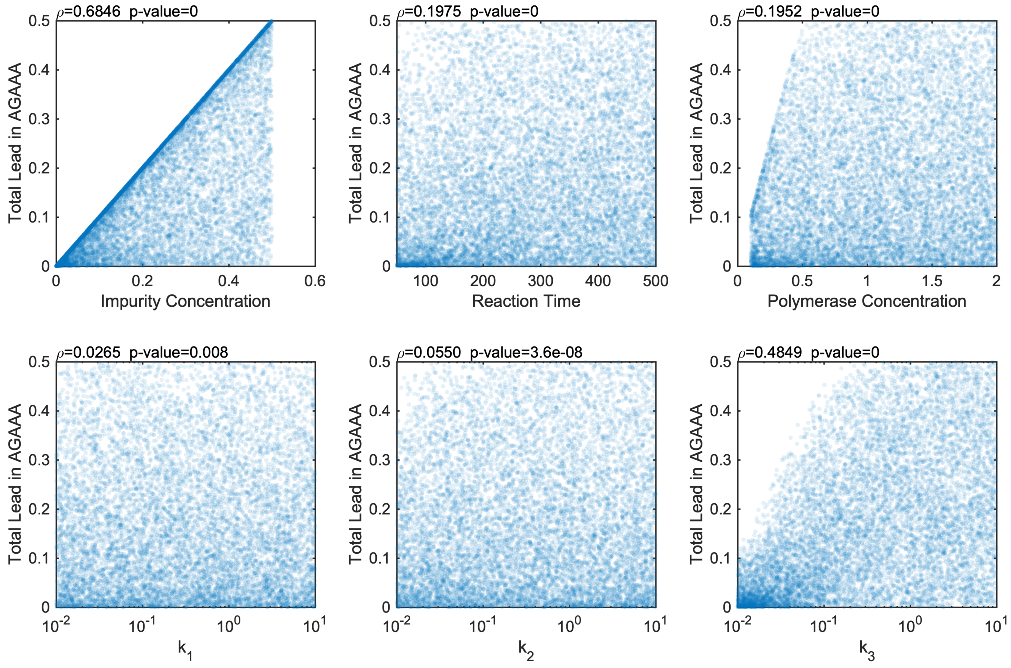

Figure S4. Scatter plot showing correlation between the six virtual sequencer parameters and total lead in sequence AGAAA. ρ: Spearman's correlation coefficient.

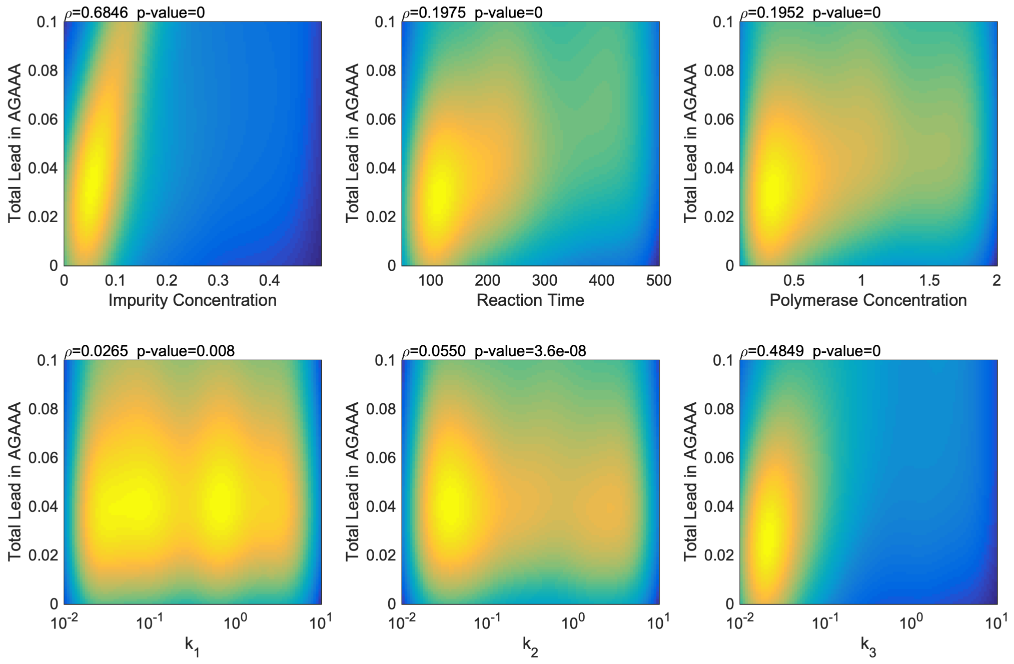

Figure S5. Heat map showing correlation between the six virtual sequencer parameters and total lead in sequence AGAAA. ρ: Spearman's correlation coefficient.

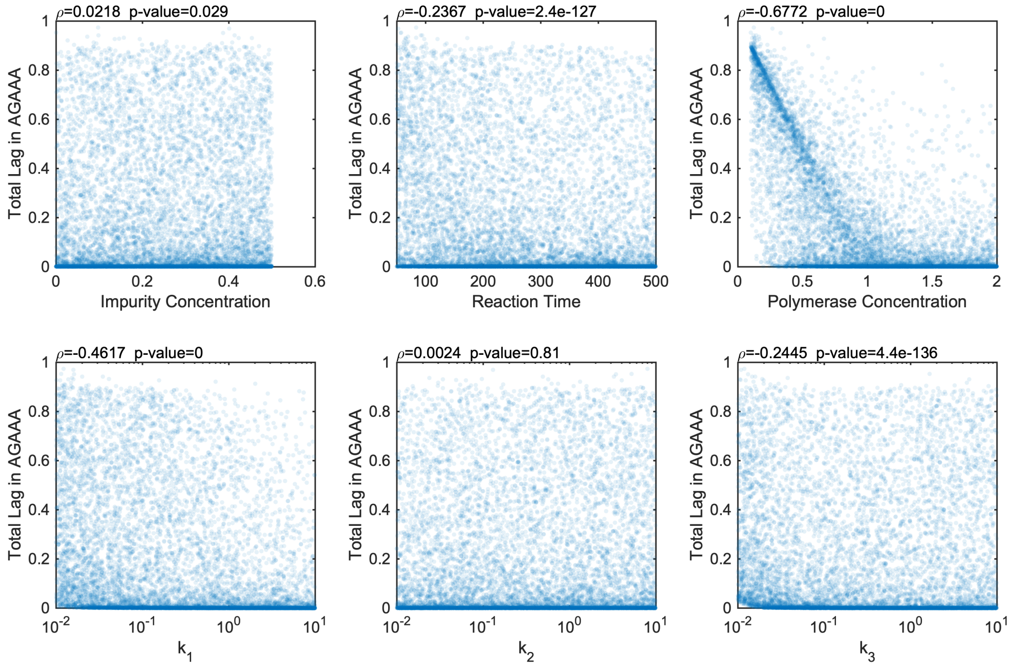

Figure S6. Scatter plot showing correlation between the six virtual sequencer parameters and total lag in sequence AGAAA. ρ: Spearman's correlation coefficient.

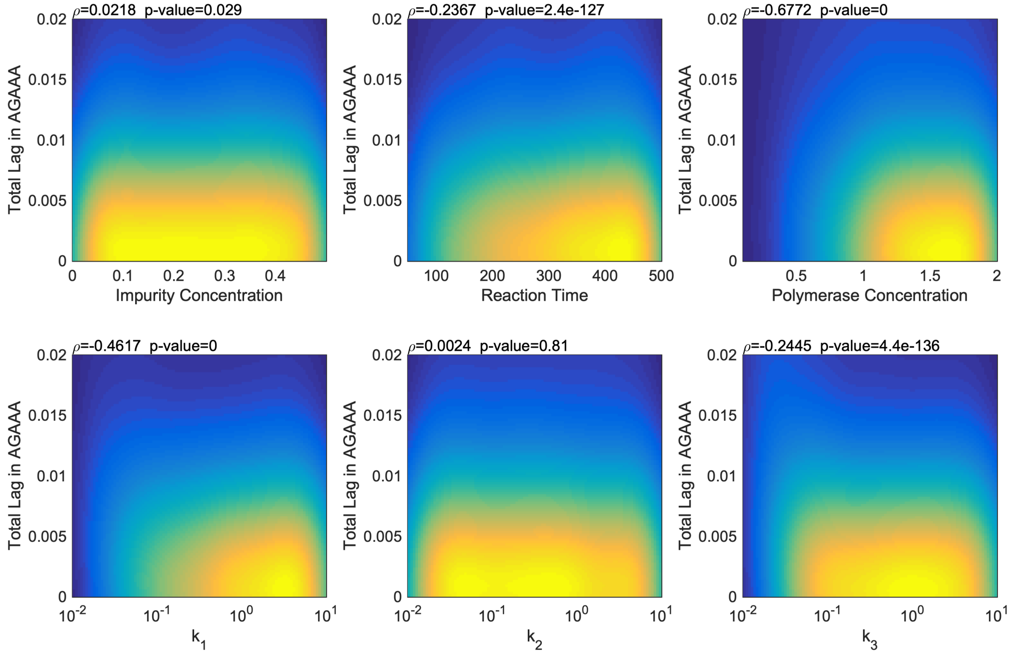

Figure S7. Heat map showing correlation between the six virtual sequencer parameters and total lag in sequence AGAAA. ρ: Spearman's correlation coefficient.

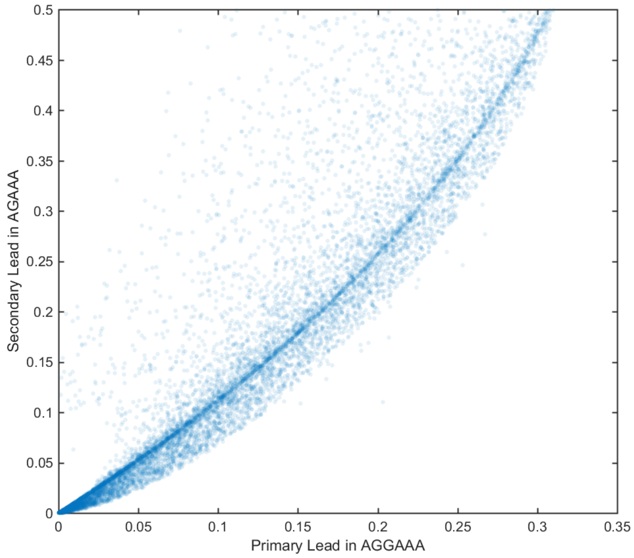

Figure S8. Scatter plot showing correlation of primary lead in sequence AGGAAA and secondary lead in sequence AGAAA.

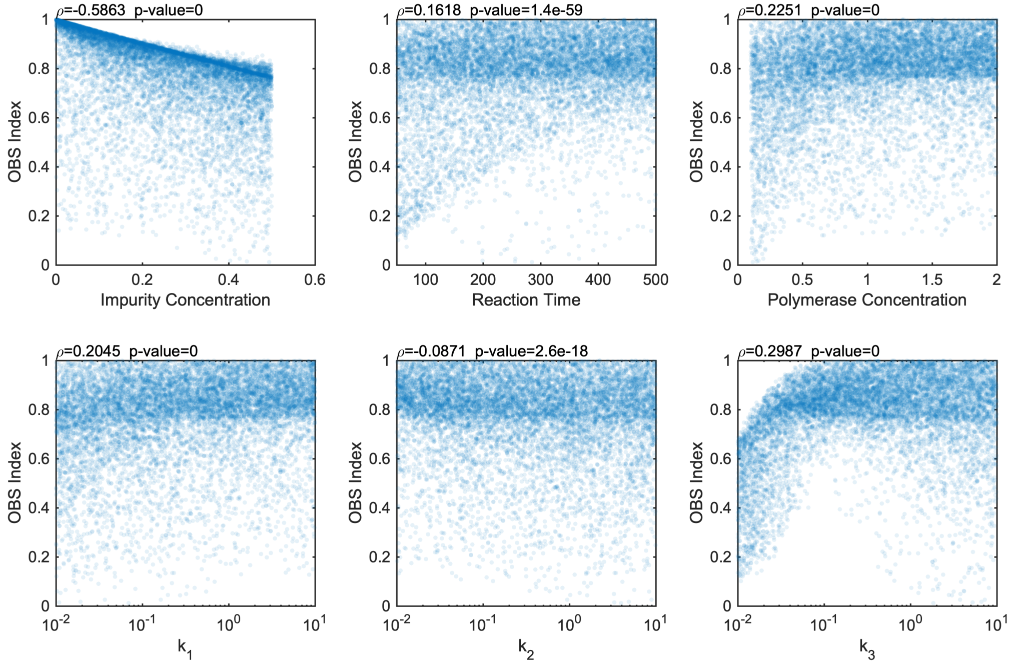

Figure S9. Scatter plot showing correlation between the six virtual sequencer parameters and the OBS index. ρ: Spearman's correlation coefficient.

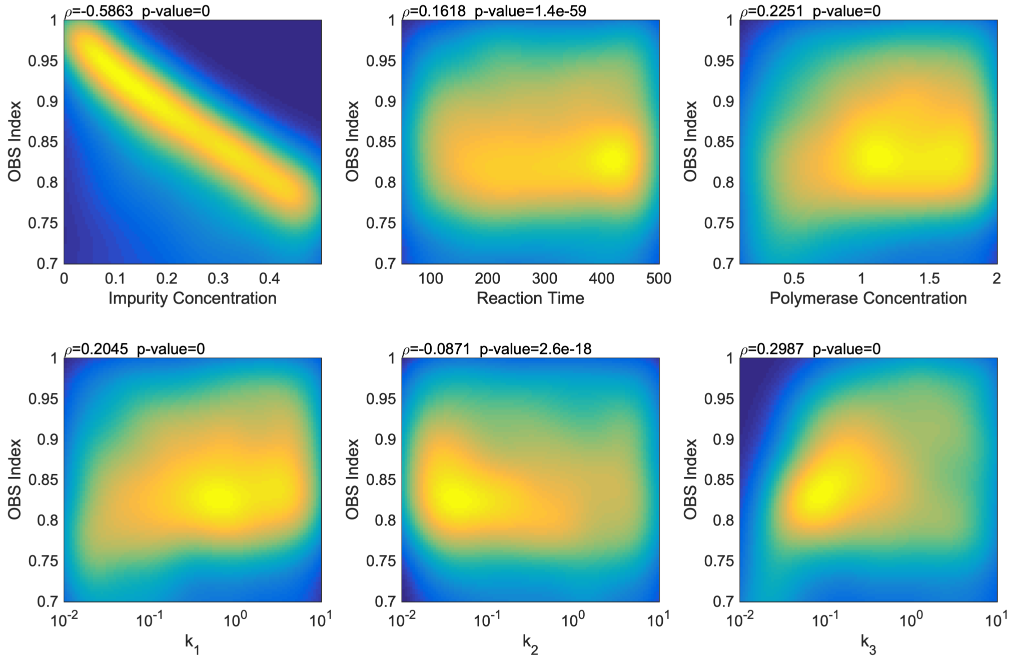

Figure S10. Heat map showing correlation between the six virtual sequencer parameters and the OBS index. ρ: Spearman's correlation coefficient.

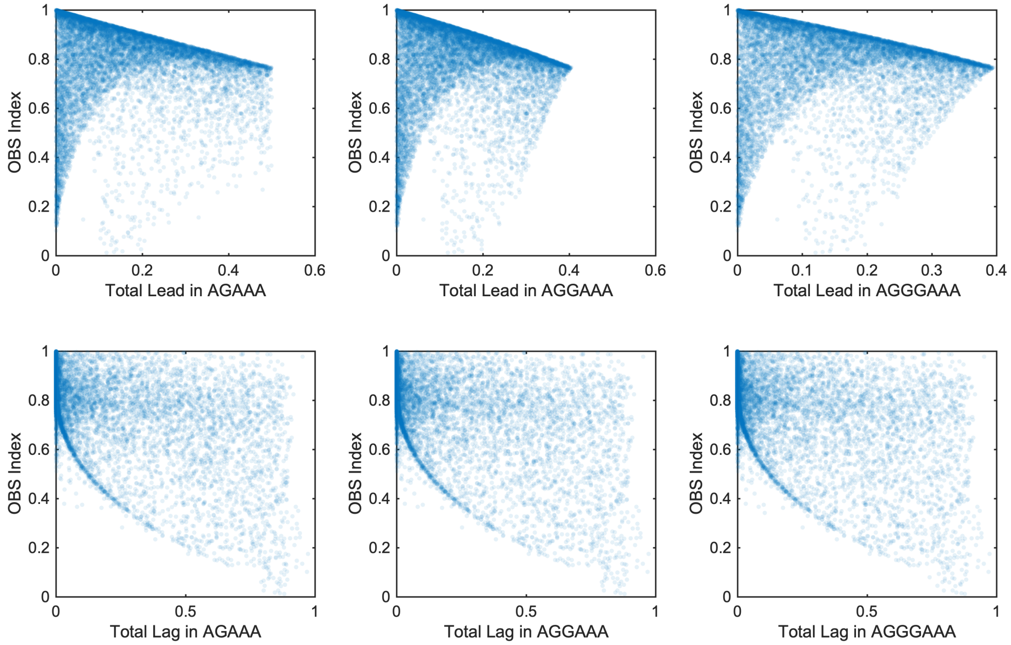

Figure S11. Correlation between dephasing parameters and OBS index.

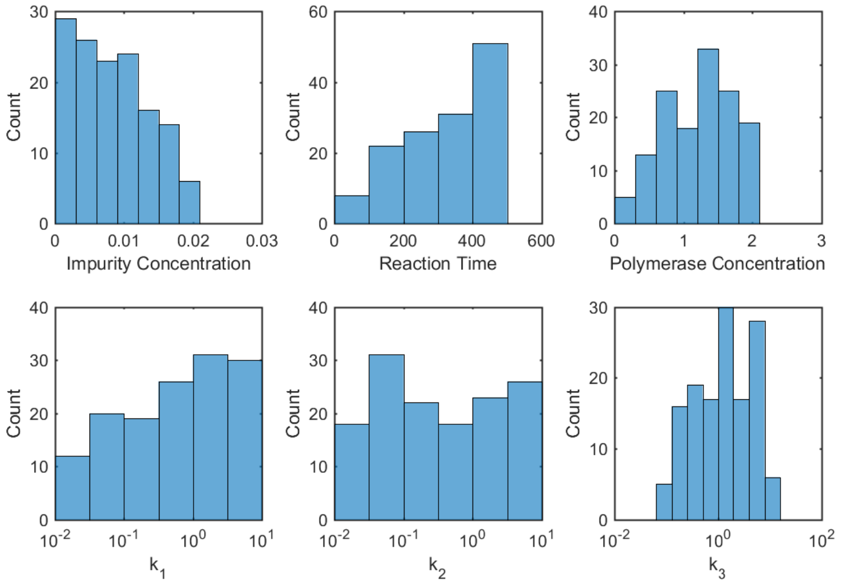

Figure S12. Distribution of virtual sequencer parameters such that ω>0.99.

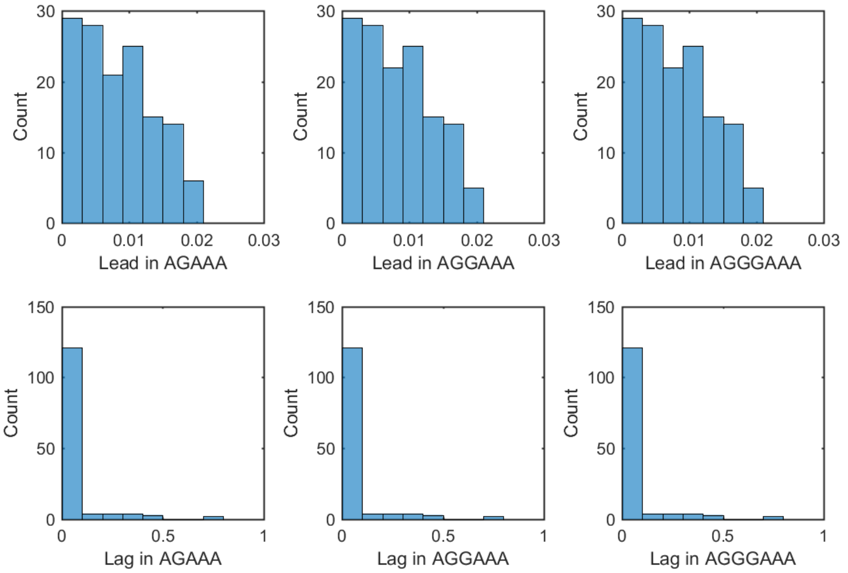

Figure S13. Distribution of dephasing parameters such that ω>0.99.

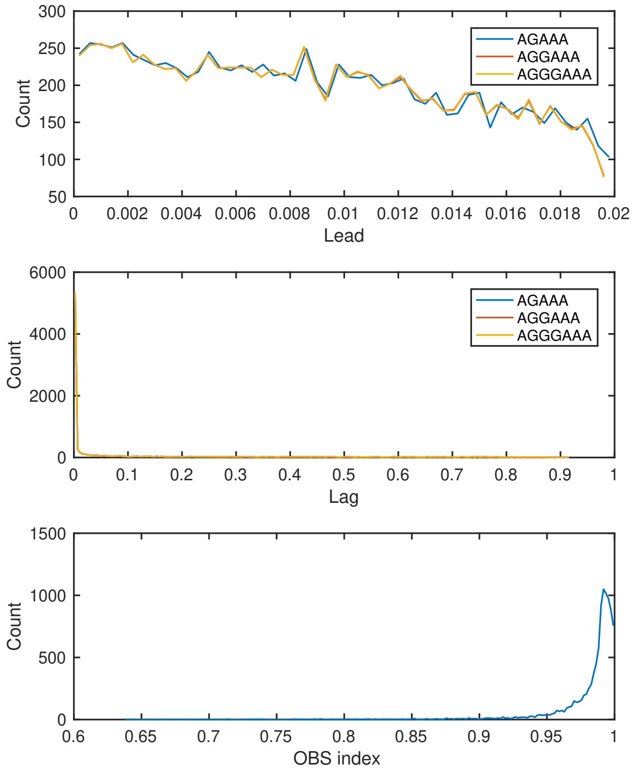

Figure S14. Distribution of total lead (top), total lag (middle) and OBS index (bottom) in Simulation 2.

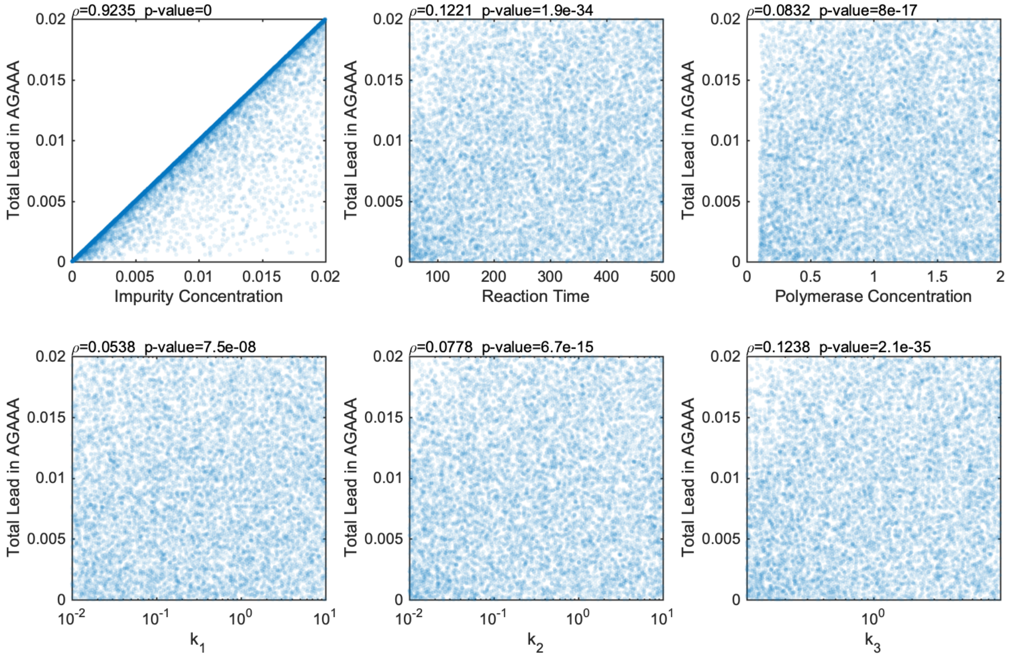

Figure S15. Scatter plot showing correlation between the six virtual sequencer parameters and total lead in sequence AGAAA in Simulation 2. ρ: Spearman's correlation coefficient.

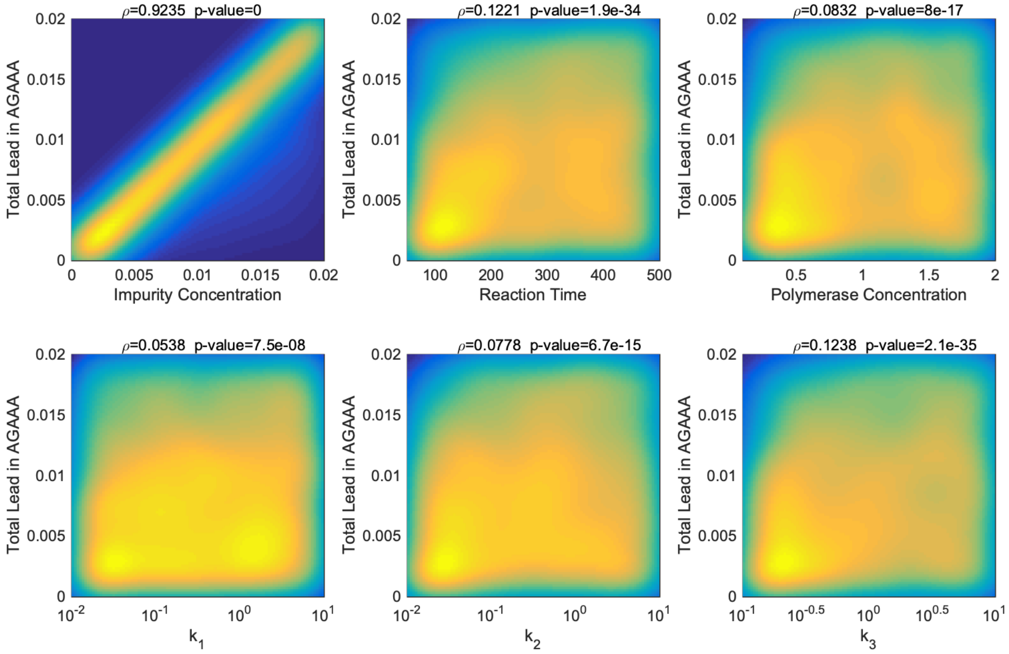

Figure S16. Heat map showing correlation between the six virtual sequencer parameters and total lead in sequence AGAAA in Simulation 2. ρ: Spearman's correlation coefficient.

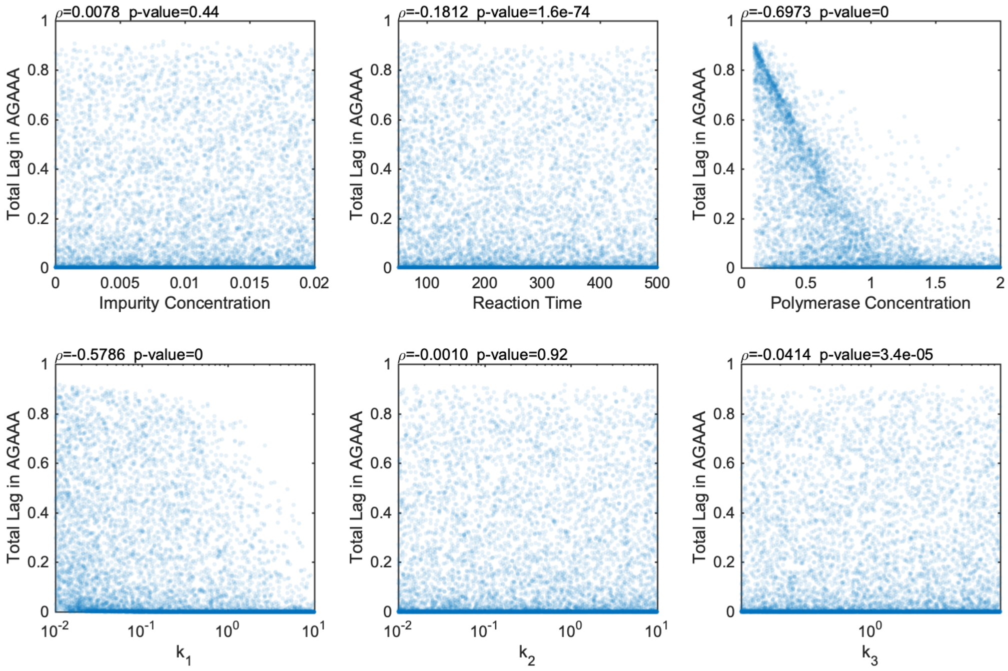

Figure S17. Scatter plot showing correlation between the six virtual sequencer parameters and total lag in sequence AGAAA in Simulation 2. ρ: Spearman's correlation coefficient.

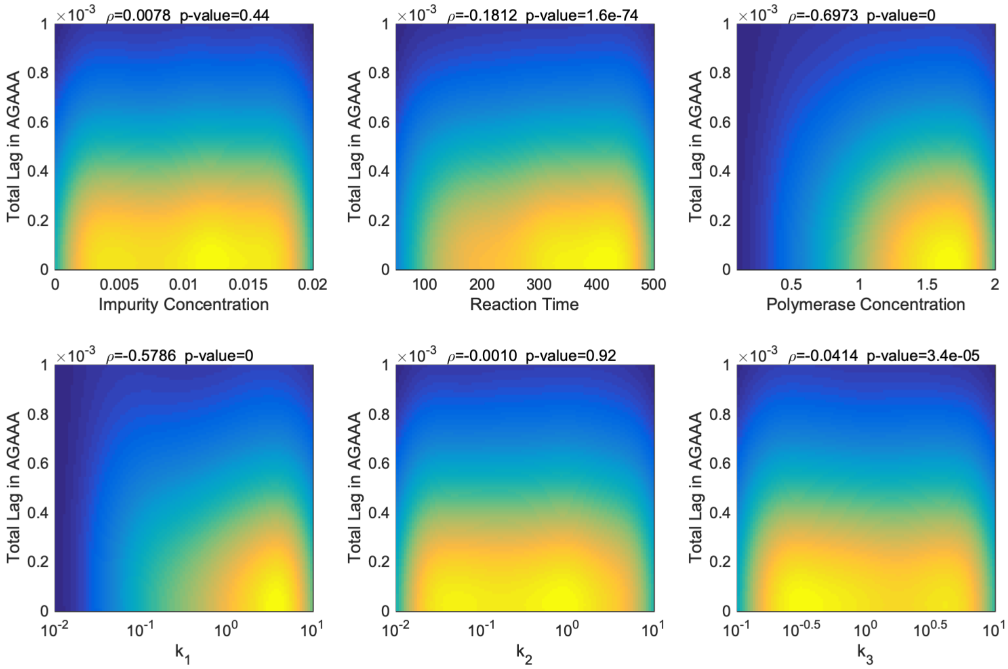

Figure S18. Heat map showing correlation between the six virtual sequencer parameters and total lag in sequence AGAAA in Simulation 2. ρ: Spearman's correlation coefficient.

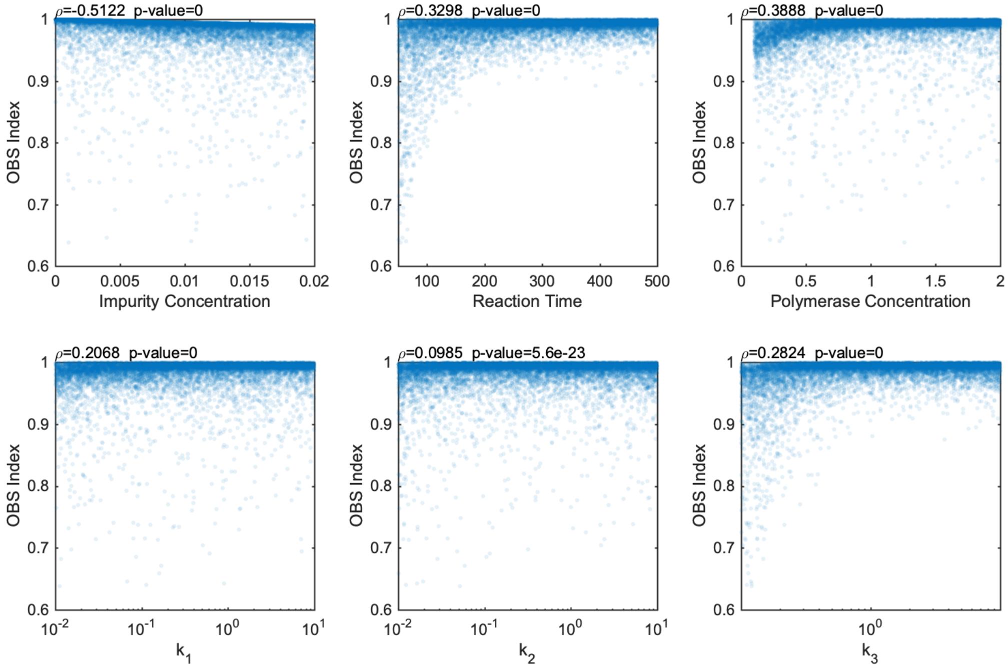

Figure S19. Scatter plot showing correlation between the six virtual sequencer parameters and OBS index in Simulation 2. ρ: Spearman's correlation coefficient.

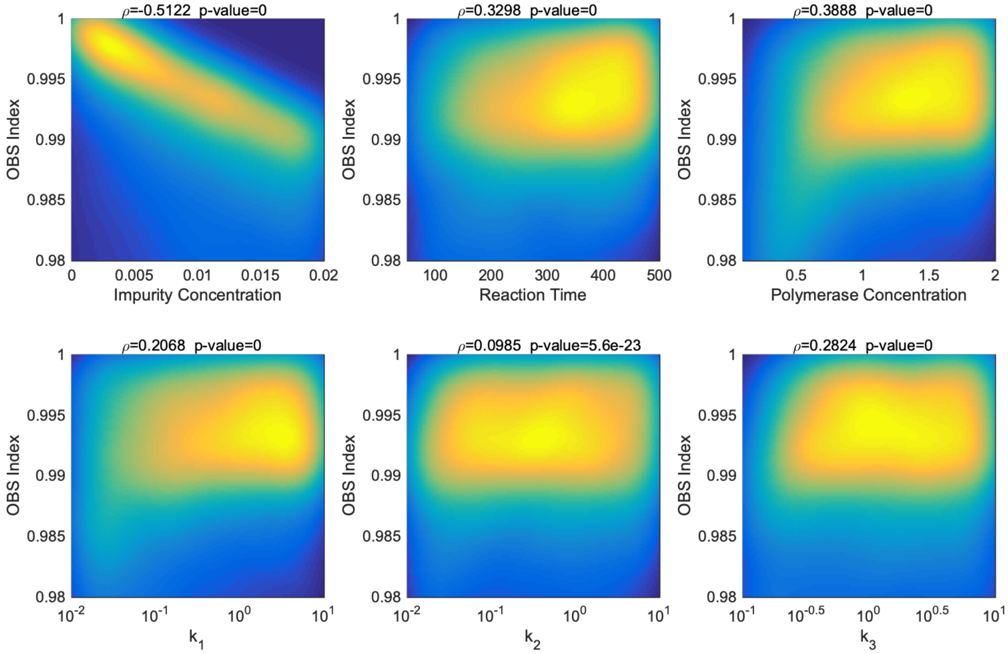

Figure S20. Heat map showing correlation between the six virtual sequencer parameters and OBS index in Simulation 2. ρ: Spearman's correlation coefficient.

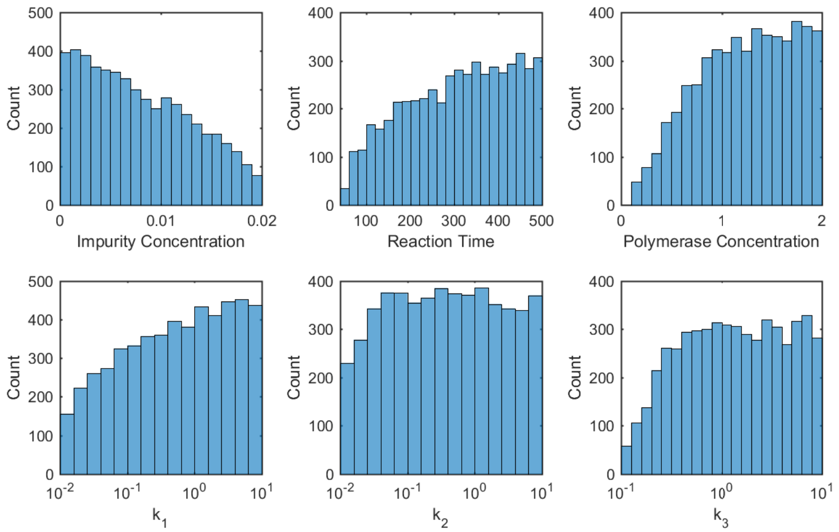

Figure S21. Distribution of virtual sequencer parameters such that ω>0.99 in Simulation 2.

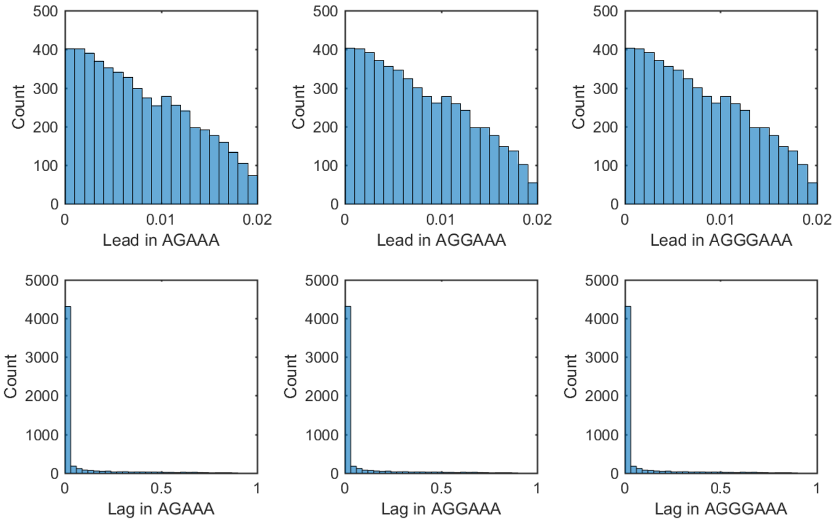

Figure S22. Distribution of dephasing parameters such that ω>0.99 in Simulation 2.

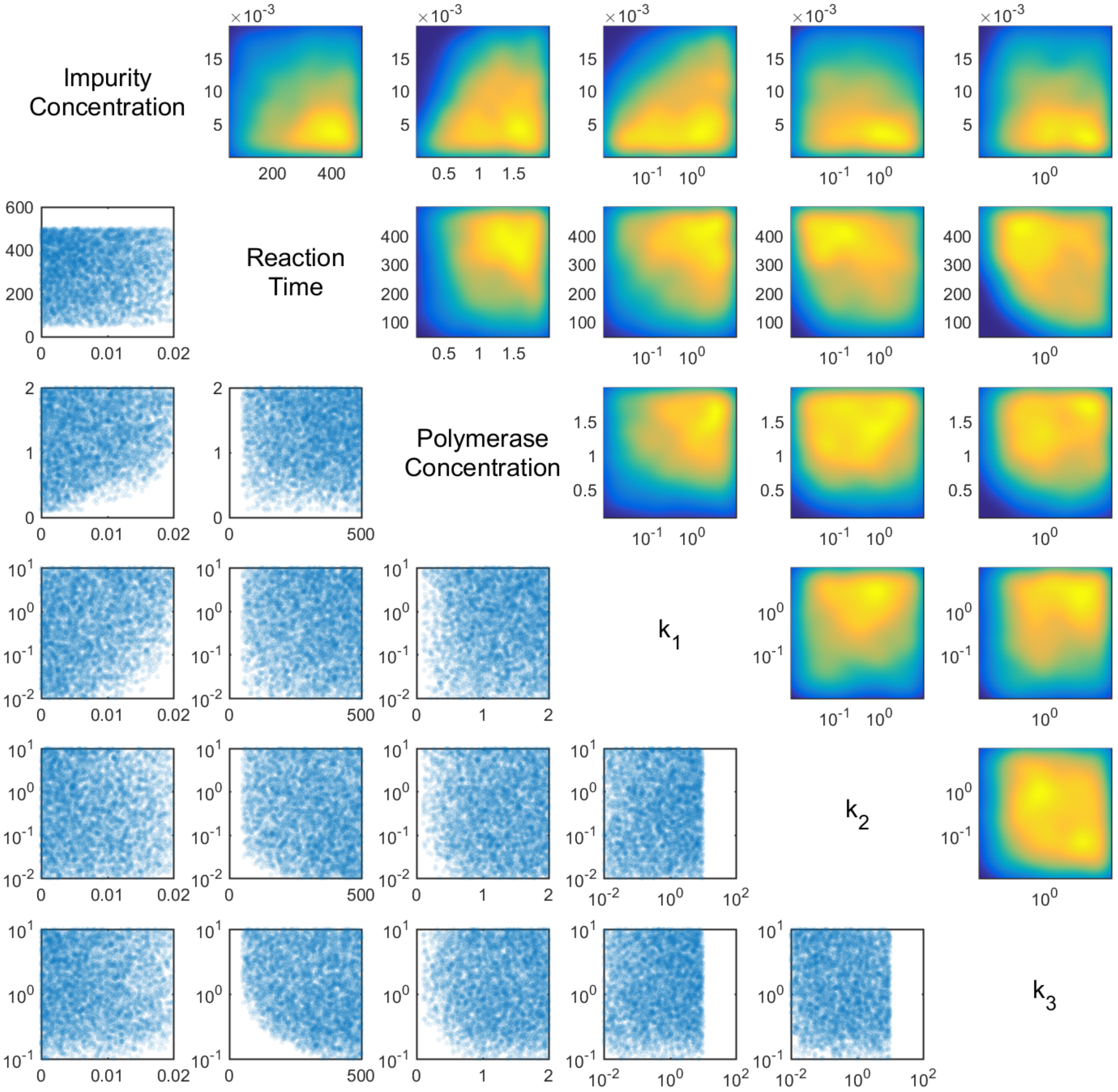

Figure S23. Pairwise correlation of virtual sequencer parameters such that $\omega > 0.99$ in Simulation 2. Above diagonal: heatmap. Below diagonal: scatter plot.

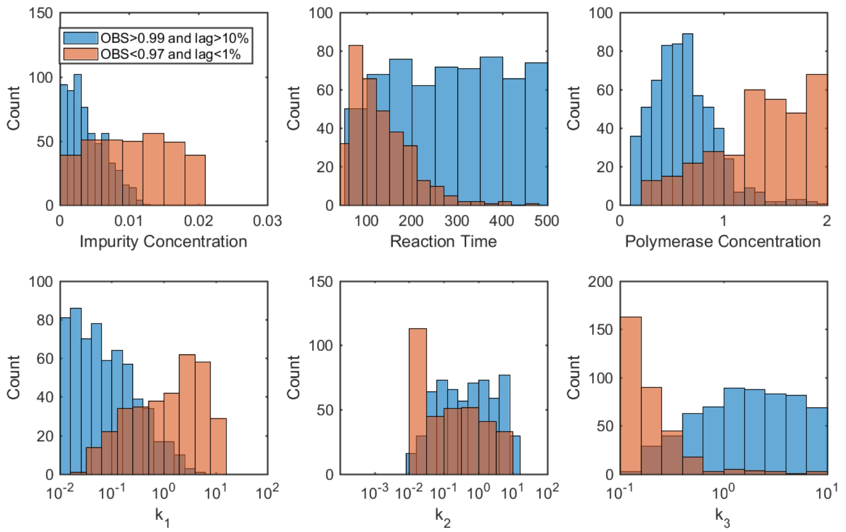

Figure S24. Distribution of two subsets of virtual sequencer parameters in Simulation 2.

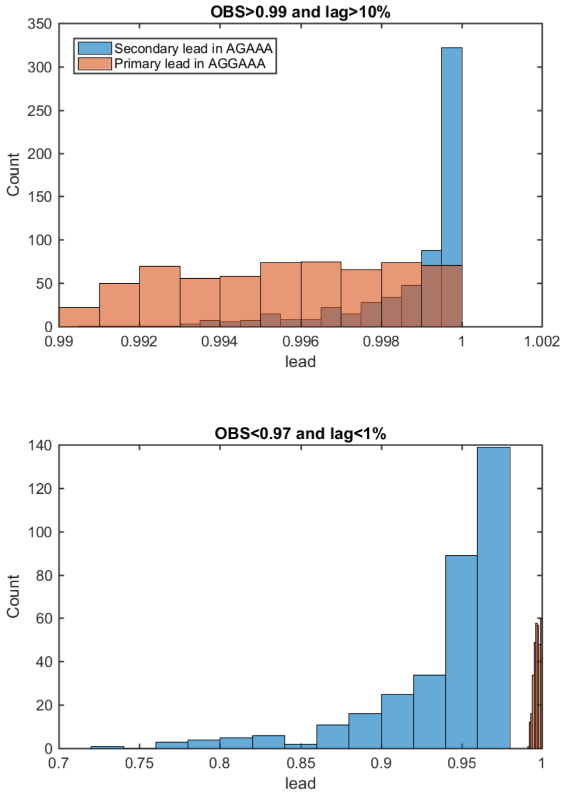

Figure S25. Distribution of two subsets of lead in Simulation 2.

Table S2. DNA templates simulated in the virtual sequencer.

Name	Genome	Position	Sequence
Seq1	*Escherichia coli*	40000-40234	CGCGCCCGGTTCGGTAATCGCTGAGTTCCACATC<br>TGCTTACCGGTGCCGCGGAAAGCCATAATTTTGT<br>CGATCTGCTCTTGTGTGCCTTCGCGCAGGAAGGT<br>GTTGAACCCGCCCGGCAACTGGTACAGCACATAG<br>GTTGGTGCCCCCAGACGTCCCAGCTCCATCCACA<br>CGGCGGCGAGAGTAACAAACCCCGCGTCCAGACC<br>ACCGTGCTCTTCAGGGATCAGCAGACTGTCG
Seq2	*Escherichia coli*	50000-50225	ATTATCCTCAGCAGTCAACCGGGTACGGACGATC<br>GCGTAACGTGGGTGAAGTCGGTGGATGAAGCCA<br>TCGCGGCGTGTGGTGACGTACCAGAAATCATGGT<br>GATTGGCGGCGGTCGCGTTTATGAACAGTTCTTG<br>CCAAAAGCGCAAAAACTGTATCTGACGCATATCG<br>ACGCAGAAGTGGAAGGCGACACCCATTTCCCGGA<br>TTACGAGCCGGATGACTGGGAAT
Seq3	*Escherichia coli*	60000-60200	ATCAGCGGCAGATCCACCAGACCTTCTGCGGGGG<br>ATGGATGCCCCCAGACGCGGGCCACATACTGCTT<br>TTTCGGCTCGCGCTCGCGGAACTGGCGTTTTAAC<br>TCCCGCTCCGCGGCTTTGGTCAGCGCCACTACAA<br>TCACGCCGCTGGTAGCCATATCCAGACGATGCAC<br>CGATTCTGCCTGCGGATAATCACGCTGAATG
Seq4	*Escherichia coli*	70000-70223	AAGCTCGCACAGAATCACTGCCAAAATCGAGGCC<br>AATTGCAATCGCCATCGTTTCACTCCATCCAAAA<br>AAACGGGTATGGAGAAACAGTAGAGAGTTGCGA<br>TAAAAAGCGTCAGGTAGGATCCGCTAATCTTATG<br>GATAAAAATGCTATGGCATAGCAAAGTGTGACGC<br>CGTGCAAATAATCAATGTGGACTTTTCTGCCGTG<br>ATTATAGACACTTTTGTTACG
Seq5	*Escherichia coli*	40000-40561	CGCGCCCGGTTCGGTAATCGCTGAGTTCCACATC<br>TGCTTACCGGTGCCGCGGAAAGCCATAATTTTGT<br>CGATCTGCTCTTGTGTGCCTTCGCGCAGGAAGGT<br>GTTGAACCCGCCCGGCAACTGGTACAGCACATAG<br>GTTGGTGCCCCCAGACGTCCCAGCTCCATCCACA<br>CGGCGGCGAGAGTAACAAACCCCGCGTCCAGACC<br>ACCGTGCTCTTCAGGGATCAGCAGACTGTCGATA<br>CCCATATCCGCCAGTGCTTTGACAAAACGTTCCG<br>GGTAGACGCTGTCACGGTCGCACTCGGCAAAATA<br>GGCCTCCCAGTTTTCGCTGGCCATCAGTTCGCGG<br>ATACCGGCGACAAACAGTTCCTGCTCATCATTTA<br>AATTAAAATCCATCTTTCAACCTCTTGATATTTT<br>GGGGGTTAATTAATCTTTCCAGTTCTGTTTCGCG<br>TCTTTAATAAAGGAGAGCGTCACCATAATGTTGA<br>CGAAGAACAGCGGGCATCCTCCGGCGATAATGGC<br>GGTTTGAATCGGTTTCAGGCCGCCGAGCGCCAGC<br>AGAACAATACCGATAATG

Seq6	*Escherichia coli*	40000-41100	CGCGCCCGGTTCGGTAATCGCTGAGTTCCACATC<br>TGCTTACCGGTGCCGCGGAAAGCCATAATTTTGT<br>CGATCTGCTCTTGTGTGCCTTCGCGCAGGAAGGT<br>GTTGAACCCGCCCGGCAACTGGTACAGCACATAG<br>GTTGGTGCCCCCAGACGTCCCAGCTCCATCCACA<br>CGGCGGCGAGAGTAACAAACCCCGCGTCCAGACC<br>ACCGTGCTCTTCAGGGATCAGCAGACTGTCGATA<br>CCCATATCCGCCAGTGCTTTGACAAAACGTTCCG<br>GGTAGACGCTGTCACGGTCGCACTCGGCAAAATA<br>GGCCTCCCAGTTTTCGCTGGCCATCAGTTCGCGG<br>ATACCGGCGACAAACAGTTCCTGCTCATCATTTA<br>AATTAAAATCCATCTTTCAACCTCTTGATATTTT<br>GGGGGTTAATTAATCTTTCCAGTTCTGTTTCGCG<br>TCTTTAATAAAGGAGAGCGTCACCATAATGTTGA<br>CGAAGAACAGCGGGCATCCTCCGGCGATAATGGC<br>GGTTTGAATCGGTTTCAGGCCGCCGAGCGCCAGC<br>AGAACAATACCGATAATGCCAACCAGAATTGACC<br>AACCGATACGCACCAGCAGAGGTGGTTCTTCACC<br>ATCGCGTACTTCGCGGCAAGTGGACATCGCCAGG<br>GTATAAGAGCAGGCGTTAACCAGCGTAACGGTGG<br>CAATAAAGCAGAGGATGAAGAAGCCCCACATGGT<br>GGCGGTGCTGAGTGGCAGAGCGGCCCAGGTTTC<br>AATGATGGCGCGCGCCACACCGTACTGTTCGATC<br>AGATTTGGAATGTTGATGATGTTTTTATCTATCA<br>ACAGCAGAGTGTTACTACCGAGTACAGTCCACAG<br>GATCCAGGTTGACGCTGTCAGCCCCAGCACCATG<br>CCGAAGCACAGTTCACGCACAGTACGACCACGGG<br>AGATGCGGGCGAGGAAGATACTCATCTGGATAGC<br>ATAAATCACCCACCATGCCCAGTAGAACACGGTC<br>CAGCCCTGCGGGAAGCCGCCTTTAGCGATGGGAT<br>CGGTATAGAACAACATGCGCGGCAGATACATCAG<br>CAACATCCCCACCGAATCGGTGAAGTAGTTCATG<br>ATGAAGCTGGCACC
lamA472	Enterobacteria phage lambda	718-1126 with a 63-bp tailing adaptor	TATCGAACAGTCAGGTTAACAGGCTGCGGCATTT<br>TGTCCGCGCCGGGCTTCGCTCACTGTTCAGGCCG<br>GAGCCACAGACCGCCGTTGAATGGGCGGATGCTA<br>ATTACTATCTCCCGAAAGAATCCGCATACCAGGA<br>AGGGCGCTGGGAAACACTGCCCTTTCAGCGGGCC<br>ATCATGAATGCGATGGGCAGCGACTACATCCGTG<br>AGGTGAATGTGGTGAAGTCTGCCCGTGTCGGTTA<br>TTCCAAAATGCTGCTGGGTGTTTATGCCTACTTT<br>ATAGAGCATAAGCAGCGCAACACCCTTATCTGGT<br>TGCCGACGGATGGTGATGCCGAGAACTTTATGAA<br>AACCCACGTTGAGCCGACTATTCGTGATATTCCG<br>TCGCTGCTGGCGCTGGCCCCGTGGTATGGCAAAA<br>ATATGATCGCATGACACGTCTGAACTCCAGTCAC<br>TGACTGATCTCGTATGCCGTCTTCTGCTTG

lamA272	Enterobacteria phage lambda	718-925 with a 64-bp tailing adaptor	TATCGAACAGTCAGGTTAACAGGCTGCGGCATTTTGTCCGCGCCGGGCTTCGCTCACTGTTCAGGCCGGAGCCACAGACCGCCGTTGAATGGGCGGATGCTAATTACTATCTCCCGAAAGAATCCGCATACCAGGAAGGGCGCTGGGAAACACTGCCCTTTCAGCGGGCCATCATGAATGCGATGGGCAGCGACTACATCCGTGAGGTAGATCGGAAGAGCACACGTCTGAACTCCAGTCACGACTGAATCTCGTATGCCGTCTTCTGCTTG

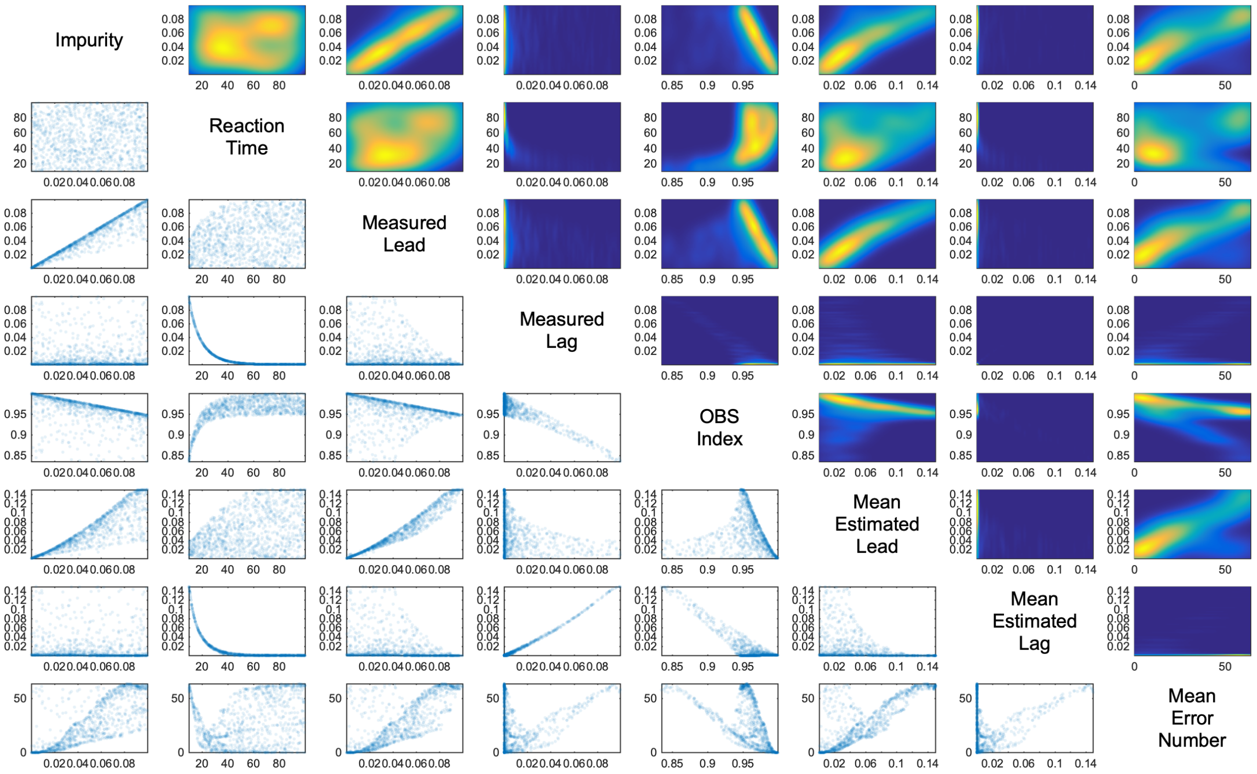

Figure S26. Pairwise correlation between the 8 parameters in the 100-cycle virtual sequencer simulation. Above diagonal: heatmap. Below diagonal: scatter plot.

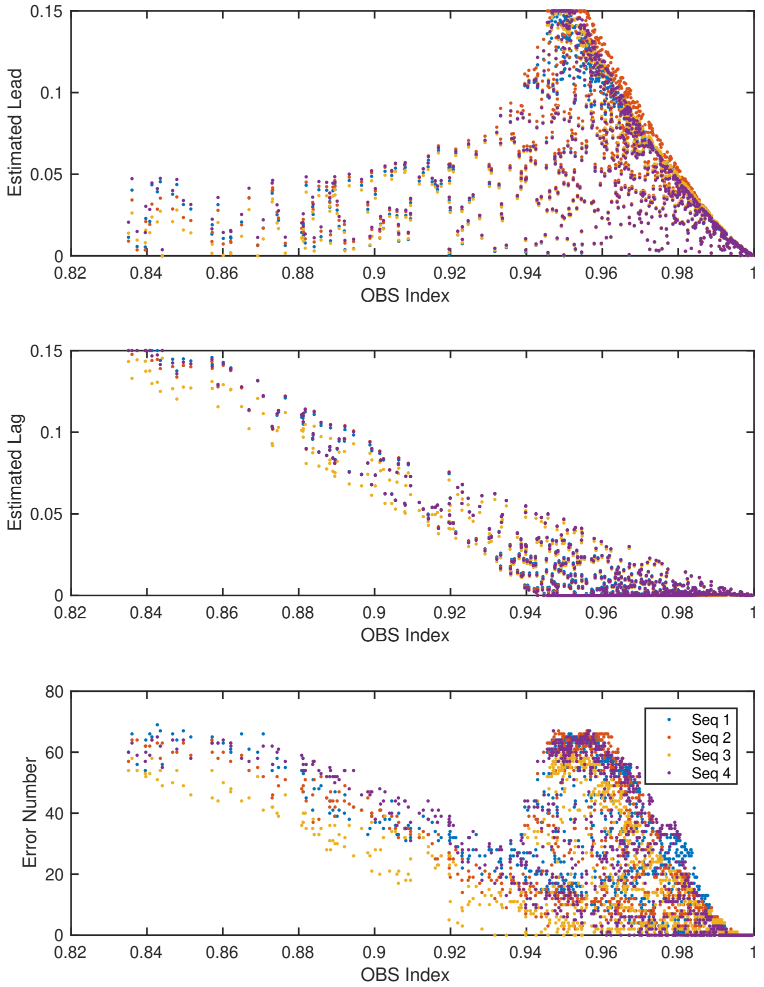

Figure S27. Correlation between the OBS index and the estimated dephasing parameters and error number after the dephasing corerction. Plotted separately.

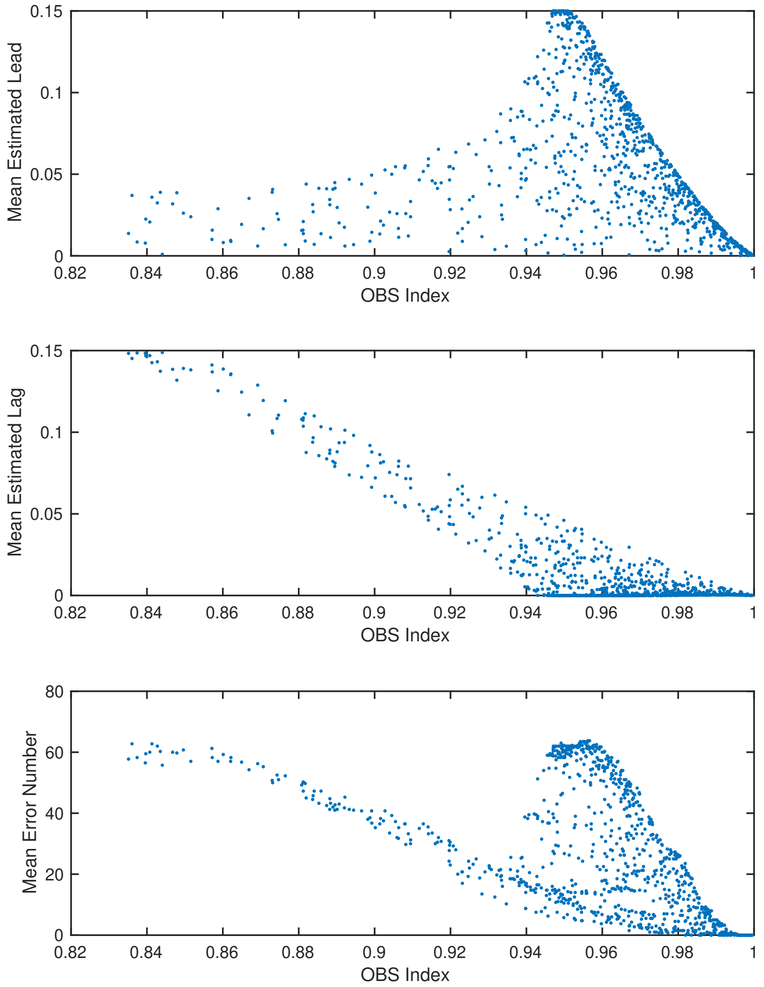

Figure S28. Correlation between the OBS index and the mean estimated dephasing parameters and error number after the dephasing corerction.

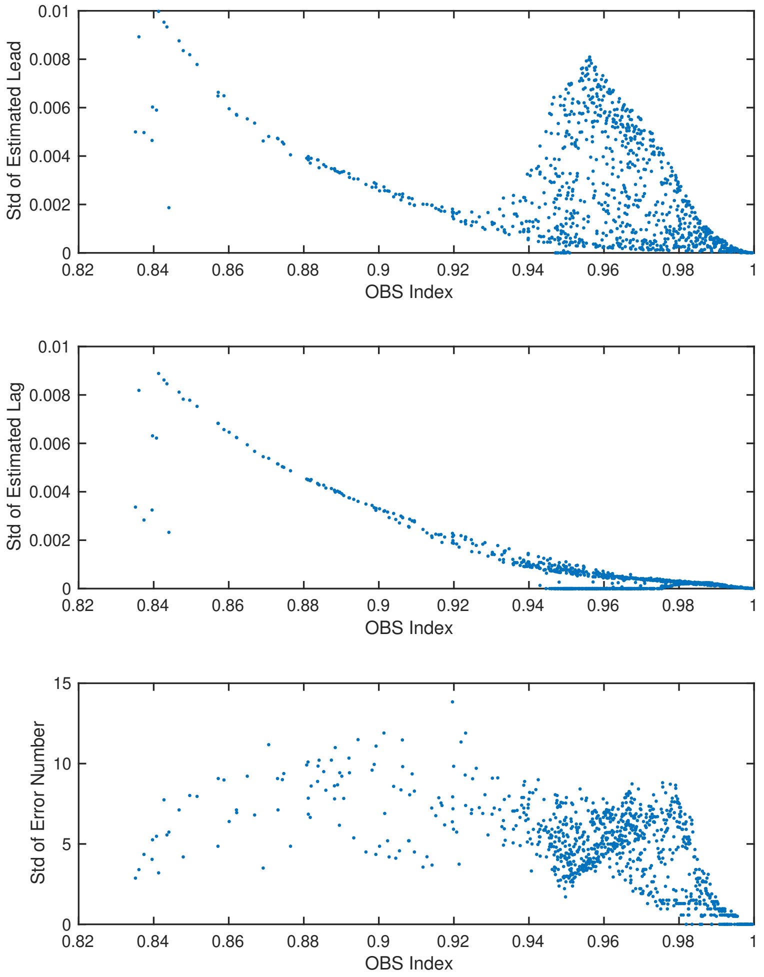

Figure S29. Correlation between the OBS index and the standard deviation of the estimated dephasing parameters and error number after the dephasing corerction.

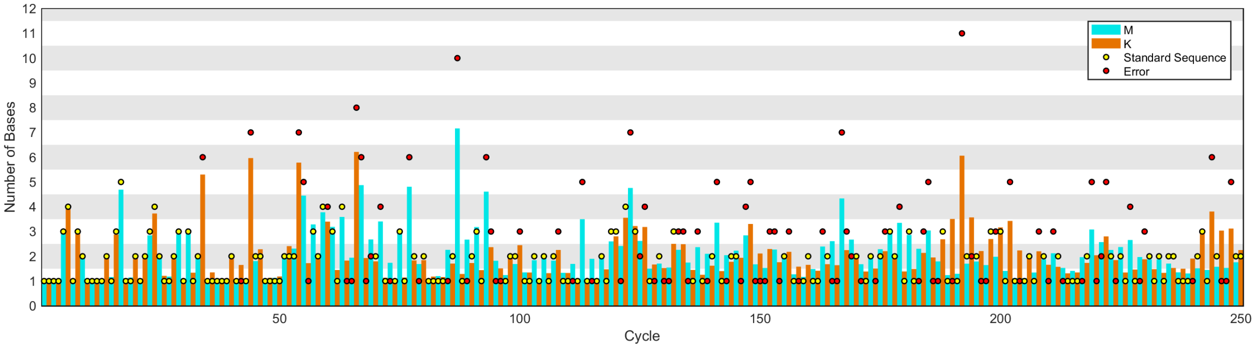

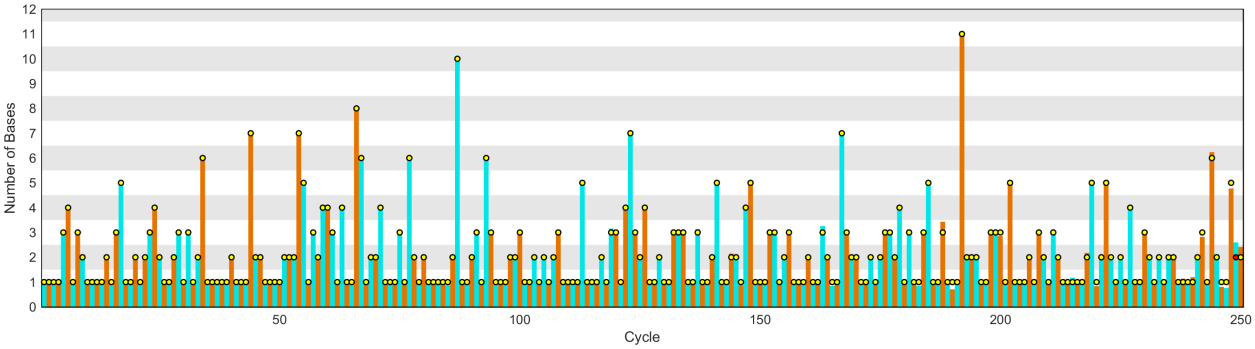

Figure S30. Simulated 250-cycle sequencing signals (top) and its dephasing-corrected signals (bottom). Impurity: 0.005; reaction time: 50.

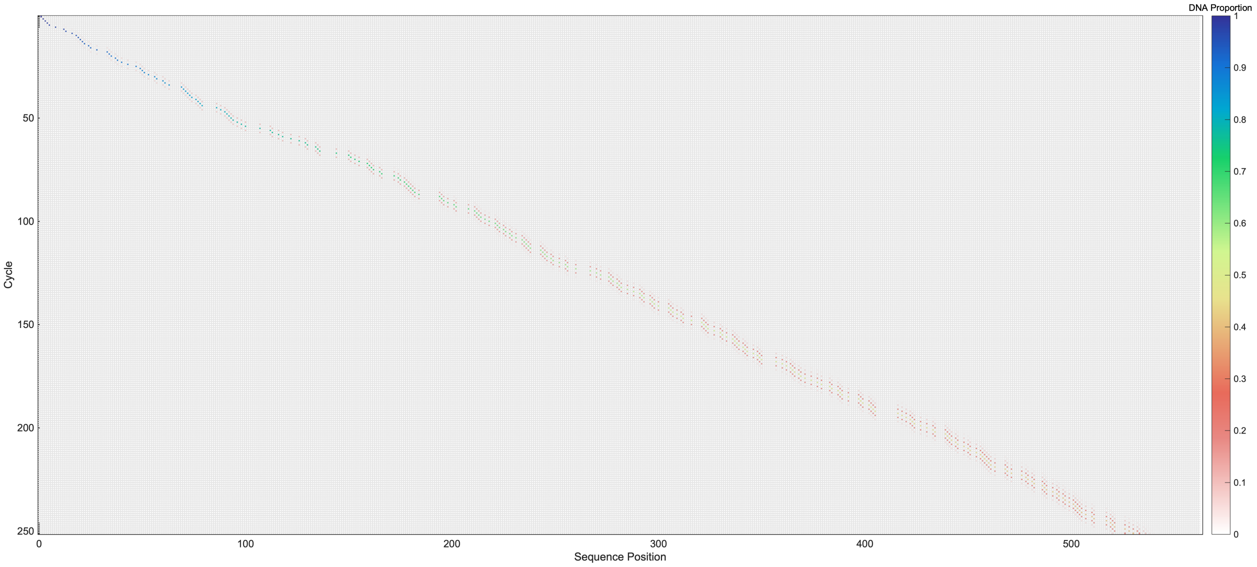

Figure S31. DNA length distribution in the 250-cycle simulation. Impurity: 0.005; reaction time: 50.

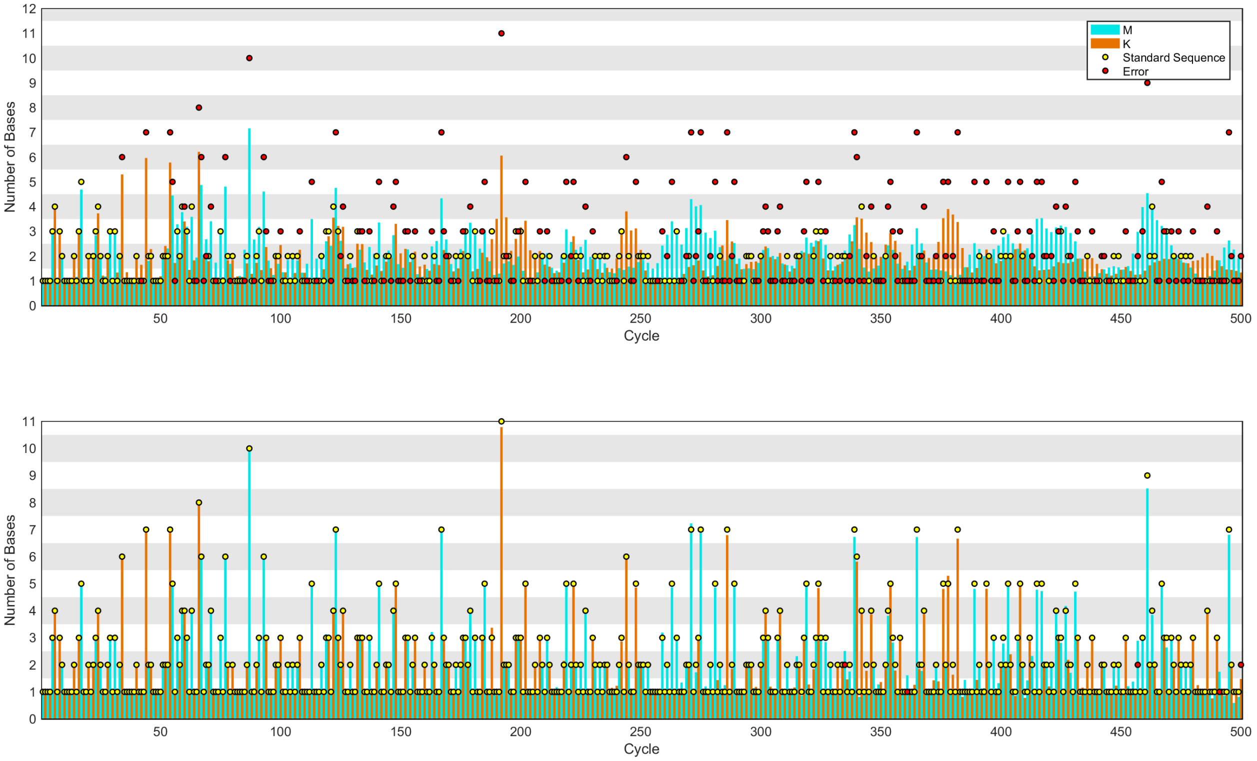

Figure S32. Simulated 500-cycle sequencing signals (top) and its dephasing-corrected signals (bottom). Impurity: 0.005; reaction time: 50.

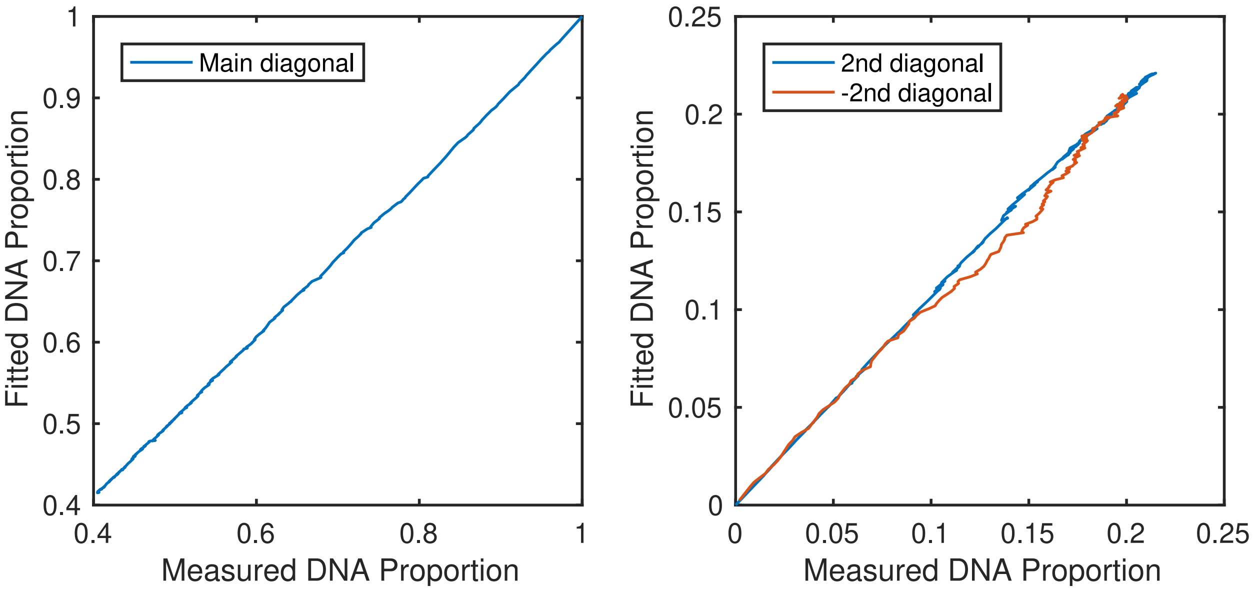

Figure S33. Comparison of DNA distribution matrix by virtual sequencer and by fitting in the 250-cycle simulation. Impurity: 0.005; reaction time: 50.

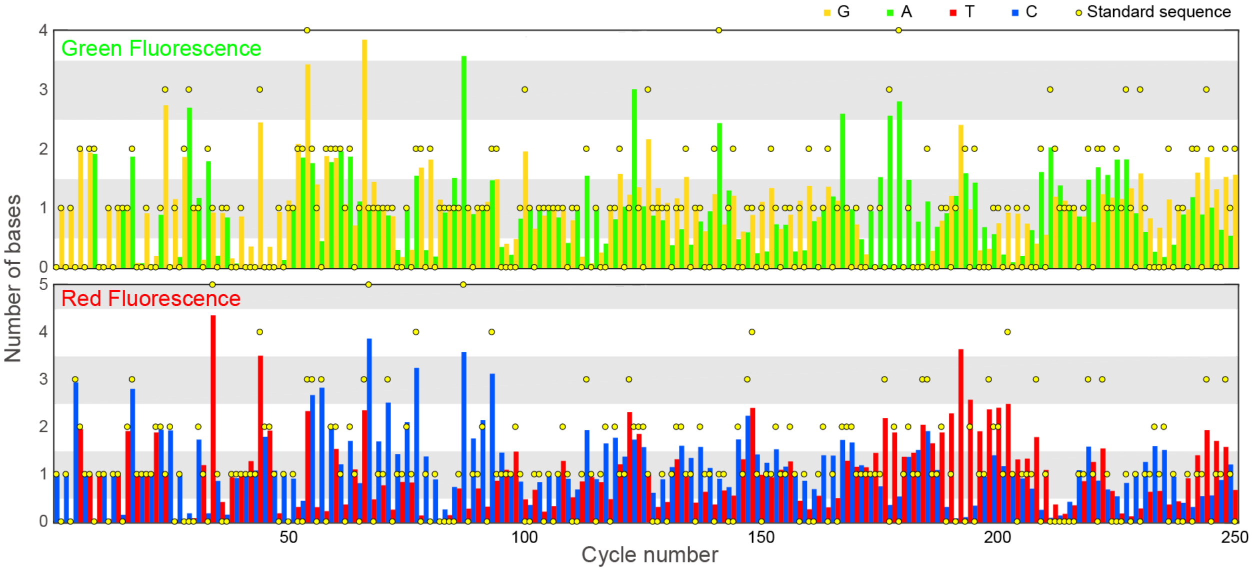

Figure S34. Simulated 250-cycle dichromatic sequencing signals. Impurity: 0.005; reaction time: 50.
